# *Aedes aegypti* in the Black Sea: recent introduction or ancient remnant?

**DOI:** 10.1186/s13071-018-2933-2

**Published:** 2018-07-06

**Authors:** Panayiota Kotsakiozi, Andrea Gloria-Soria, Francis Schaffner, Vincent Robert, Jeffrey R. Powell

**Affiliations:** 1 Yale University, 21 Sachem Street, New Haven, CT 06520-8105 USA; 2 The Connecticut Agricultural Experiment Station, 123 Huntington Street, New Haven, CT 06504 USA; 3 Francis Schaffner Consultancy, Lörracherstrasse 50, 4125 Riehen, Switzerland; 4 National Centre for Vector Entomology, Institute of Parasitology, Vetsuisse Faculty, University of Zurich, Winterthurerstrasse 266a, 8057 Zurich, Switzerland; 5 MIVEGEC Unit, IRD, CNRS, University Montpellier, Montpellier, France

**Keywords:** Yellow fever mosquito, Evolutionary history, Phylogeny, Arbovirus vector, Population genetics, SNPs, Microsatellites

## Abstract

**Background:**

The yellow fever mosquito *Aedes aegypti* transmits viral diseases that have plagued humans for centuries. Its ancestral home are forests of Africa and ~400–600 years ago it invaded the New World and later Europe and Asia, causing some of the largest epidemics in human history. The species was rarely detected in countries surrounding the Mediterranean Sea after the 1950s, but during the last 16 years it re-appeared in Madeira, Russia and in the eastern coast of the Black Sea. We genotyped *Ae. aegypti* populations from the Black Sea region to investigate whether this is a recent invasion (and if so, where it came from) or a remnant of pre-eradication populations that extended across the Mediterranean. We also use the Black Sea populations together with a world reference panel of populations to shed more light into the phylogeographical history of this species.

**Results:**

Microsatellites and ~19,000 genome-wide single nucleotide polymorphisms (SNPs) support the monophyletic origin of all populations outside Africa, with the New World as the site of first colonization. Considering the phylogenetic relationships, the Black Sea populations are basal to all Asian populations sampled. Bayesian analyses combined with multivariate analyses on both types of markers suggest that the Black Sea population is a remnant of an older population. Approximate Bayesian Computation Analysis indicates with equal probability, that the origin of Black Sea populations was Asia or New World and assignment tests favor the New World.

**Conclusions:**

Our results confirmed that *Ae. aegypti* left Africa and arrived in New World ~500 years ago. The lineage that returned to the Old World and gave rise to present day Asia and the Black Sea populations split from the New World approximately 100–150 years ago. Globally, the Black Sea population is genetically closer to Asia, but still highly differentiated from both New World and Asian populations. This evidence, combined with bottleneck signatures and divergence time estimates, support the hypothesis of present day Black Sea populations being remnants of older populations, likely the now extinct Mediterranean populations that, consistent with the historic epidemiological record, likely represent the original return of *Ae. aegypti* to the Old World.

**Electronic supplementary material:**

The online version of this article (10.1186/s13071-018-2933-2) contains supplementary material, which is available to authorized users.

## Background

*Aedes aegypti* bears the official common name of “the yellow fever mosquito”, although today it is more feared as the major vector of viruses causing dengue, chikungunya and Zika fevers. There is little doubt that the origin of *Ae. aegypti* is Africa. This is consistent with ecology, biogeography, genetics and historical records (for a review see [1] and references within). The ancestral form of *Ae. aegypti* still breeds in tropical forests in sub-Saharan Africa, where its larvae are found inside tree holes and females prefer non-human blood meals [2, 3]. At some point during its evolutionary history, this forest-breeding mosquito became adapted to human habitats, with larvae breeding in man-made containers and adult females favoring human blood meals. This “domesticated” form of the mosquito spread with man around the world to tropical and subtropical regions [1, 4]. These two forms have been given subspecific names, *Ae. aegypti formosus* (*Aaf*) for the ancestral forest form and *Ae. aegypti aegypti* (*Aaa*) for the domestic form.

The distribution of *Ae. aegypti* outside Africa has been dynamic, as expected of an invasive human commensal [5]. After its global geographical expansion, one of the major changes was its disappearance from the Mediterranean basin, where it was once widely established and responsible for outbreaks of yellow fever and dengue in the 19th and 20th centuries [6, 7]. *Aedes aegypti* has rarely been detected in countries surrounding the Mediterranean Sea after the 1950s, with sporadic reports coming from Italy, Israel and Turkey (for details see [7] and references within). This disappearance has been attributed to a combination of DDT use aimed at malaria-transmitting *Anopheles*, vector control measures in response to the Greek dengue epidemic, colder winters, and perhaps more importantly, improvements in sanitation, indoor plumbing in particular [8, 9].

After an apparent 50 year absence from Europe, *Ae. aegypti* was detected in 2001 in the area of Sochi, Russia and then in 2005 on the Portuguese island of Madeira [10]. By 2008, it had re-populated the eastern coast of the Black Sea, with populations reported in Georgia, Russia [11, 12] and later (2015) from Turkey [13].

The current presence of *Ae. aegypti* in the Black Sea area may represent a new introduction from *Ae. aegypti* endemic areas (the Americas, Africa or Asia), or a cryptic population related to the *Ae. aegypti* that once populated the Mediterranean basin.

Here we present genetic data (microsatellites and SNPs) on populations of *Ae. aegypti* from Turkey and Georgia. We examine the genetic diversity and differentiation patterns among these populations and compare them with the known established populations of *Ae. aegypti* worldwide, with the goals of (i) determining whether present-day Black Sea populations are recent invasions or re-emergence of older cryptic populations; (ii) reconstructing the most probable biogeographical scenario for the invasion to the Black Sea region; and (iii) revealing the evolutionary relationships of the Black Sea populations with the known established populations in the New World, Asia and Africa.

## Methods

### Mosquito collections, DNA extraction and genotyping

**Fig. 1:**
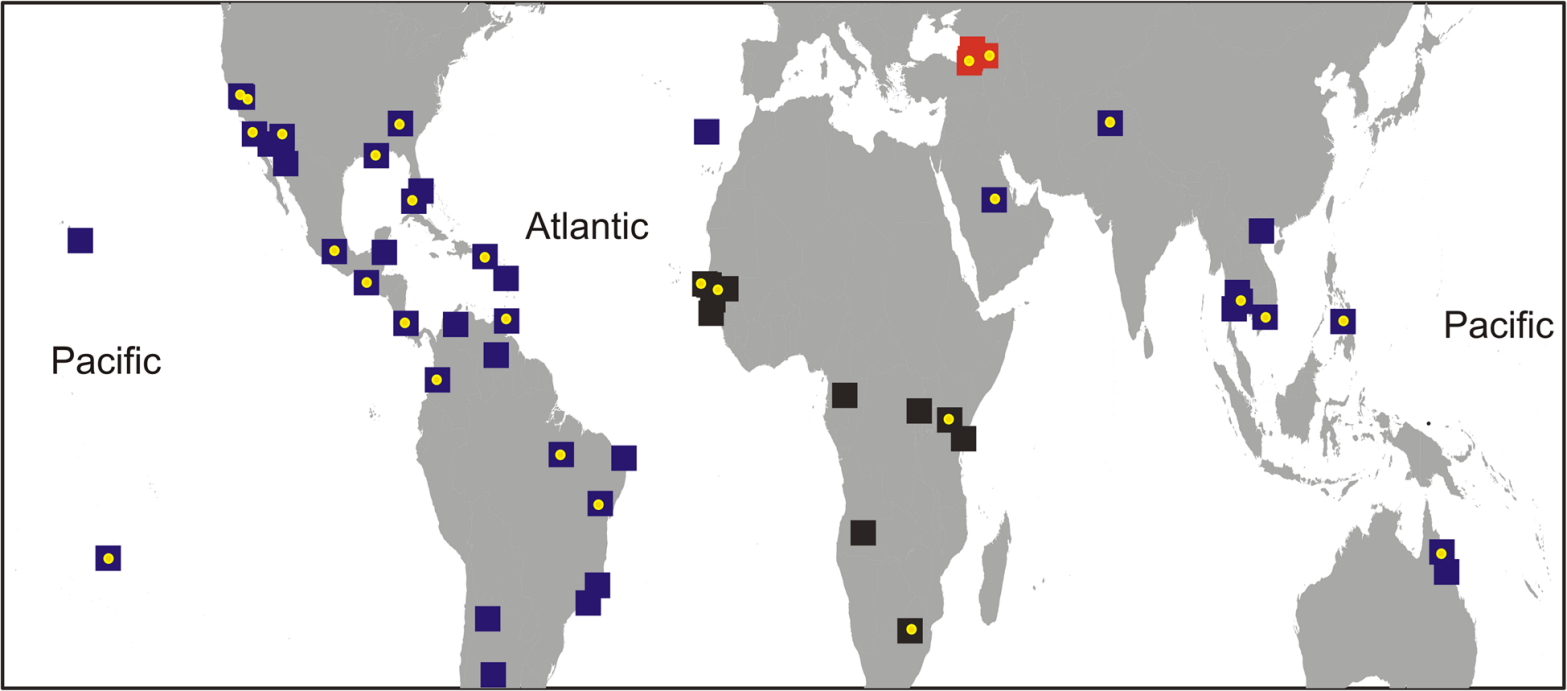
Populations of *Ae. aegypti* used in the study. The Black Sea populations that are the focus of this study are indicated with red and the reference panel of populations used to identify their origin(s) are indicated with blue (*Ae. ae. aegypti*) and black (*Ae. ae. formosus*). All populations have microsatellite data and those marked with yellow dot also have SNP data

Two different datasets of genetic markers were used in this study: (i) twelve previously published microsatellite loci [A1, B2, B3, A9 (tri-nucleotide repeats), and AC2, CT2, AG2, AC4, AC1, AC5, AG1, and AG4 (di-nucleotide repeats)] [14, 15]; and (ii) a panel of ~25,000 SNPs [16]. The complete microsatellite dataset consisted of 1795 individuals from 56 populations (Fig. 1), while the SNP dataset consisted of 306 individuals from 30 populations (for details see Additional file 1: Table S1).

The Black Sea region is represented by samples from Turkey and Georgia. Two locations were sampled from Turkey (~50 km apart) and three locations from Georgia (inland: 2 locations; 15 individuals in total; and coast: 23 individuals). Since the sample size of the two inland Georgia localities was small (6 and 9 individuals), the preliminary analysis indicated that they form one homogenous group with low differentiation among them, and due to their geographical proximity (within ~10 km), these samples were pooled and treated as a single population sample of 15 individuals labeled as Georgia-inland.

For both datasets, total DNA was extracted using the DNeasy Blood and Tissue kit (Qiagen, Hilden, Germany) according to the manufacturer instructions, with an additional RNAse A (Qiagen) step. DNA samples were stored at -20 °C until further analysis. For the microsatellites dataset, individual mosquitoes were genotyped as described in [14]. Microsatellite alleles were scored using GENEMAPPER version 4.0 (Applied Biosystems). The SNPs dataset included the 25,589 SNPs as they genotyped in the *Ae. aegypti* SNP-Chip [16] of Affymetrix. Briefly, genomic DNA (~200 ng per sample) was sent to the Functional Genomics Core at the University of North Carolina, Chapel Hill, for hybridization and production of genotypes. The Affymetrix Genotyping Console and the R package SNPolisher v1.4 (both from Affymetrix, Inc., Santa Clara, CA, USA) were used to generate and process genotype calls.

Raw data are available from VectorBase.org, Project ID VBP0000269 (microsatellite and SNPs).

### Genetic diversity and differentiation

#### Microsatellite loci

Within-population deviations from Hardy-Weinberg equilibrium (HWE) were estimated using the exact HWE test in GENEPOP v.4.5.1 [17, 18]. The test was run with 10,000 dememorizations, 1,000 batches and 10,000 iterations per batch. Bonferroni correction (α = 0.05) was applied to the resulting matrix of HWE. Number of alleles, allelic frequencies, and average observed (Ho) and expected (He) heterozygosities were estimated using GenAlEx [19]. Allelic richness (AR) and private allelic richness (Np) were calculated in HPRARE [20, 21] using rarefaction and assuming 30 genes. The non-parametric Kruskal Wallis test was used to test for significant differences on Ho and allelic richness between different groups of populations.

#### SNP loci

Pairwise genetic distances (*F*_ST_) between all pairs of populations and their significance were calculated in the SNP dataset with Arlequin v3.5.1.2 [22], using 1000 permutations.

### Genetic structure

Many of the populations included in the microsatellite dataset have been studied previously [14, 23–26] and here are used as a worldwide reference panel to accurately identify the origin(s) of the Black Sea populations and their genetic affinities with the other *Ae. aegypti* populations throughout the world.

Geographical population structure was evaluated using the Bayesian clustering method implemented in the software STRUCTURE v.2.3 [27] for the microsatellite dataset, and the fastSTRUCTURE [28] for the SNP dataset. Prior to the implementation of the fastSTRUCTURE analysis, the SNP dataset was filtered to exclude highly linked SNPs, using the *--*indep-pairwise 50 10 0.3 parameter in PLINK [29].

Both methods identify genetic clusters and assign individuals to clusters with no *a priori* information of sampling location. For the STRUCTURE analysis the most likely number of clusters (*K*), was determined by conducting 20 independent runs for each *K* = 1 to the maximum number of populations included in the analysis. Each run assumed an admixture model and independent allele frequencies (λ set to 1), using a burn-in value of 100,000 iterations followed by 500,000 repetitions. The optimal number of *K* clusters was determined using the Δ*K* method of Evanno et al. [30], using the online version of STRUCTURE HARVESTER v.0.6.94 [27].

Since there is increasing evidence [31, 32] that STRUCTURE analysis is severely affected by uneven sampling, which could lead to wrong inferences on hierarchical structure, each population was represented by a random subsample of 34 individuals (or less if not available) in the microsatellite dataset and by 12 random individuals (or less if not available) in the SNP dataset, to match the sizes of the Black Sea samples (Additional file 1: Table S1). Following the same reasoning and given that the Black Sea region is underrepresented (four populations) compared with the other geographical groups, we performed the genetic structure analyses of both the microsatellite and the SNPs datasets twice: (i) using all the populations available; and (ii) by randomly selecting (multiple times) four populations each from Africa, New World and Asia-Pacific regions. Results from the analyses of all datasets were summarized and plotted using the online version of CLUMPAK [33].

To complement the genetic structure analyses, we performed Principal Components Analysis (PCA) and Discriminant Analysis of Principal Components (DAPC), using the R packages *ade4* [34], *LEA* [35] and *ADEGENET* [36] in R v.3.4.4 [37] on both datasets.

### Assignment test

To test the degree of assignment of any individual mosquito to a specific population of origin or geographical group, we used the program GeneClass2 v2.0 [38]. Assignment tests were performed on both datasets using the Rannala & Mountain criterion [39] and the Monte Carlo resampling algorithm of Paetkau et al. [40] (*n* = 1000) for level of significance 0.05. Due to limitations on the number of loci that can be analyzed by the GeneClass2 software, five independent analyses were run using 4000 randomly selected SNPs. Self-assignment tests on the reference population panel resulted in 97.7% of the individuals correctly assigned to their population of origin.

### Bottleneck effect

We tested for evidence of population bottleneck events using two methods, as implemented in BOTTLENECK [41]. In the first method, the distribution of the expected heterozygosity from the observed number of alleles is calculated for each population and locus, under the assumption of mutation-drift equilibrium; the second method is based on allele frequency distributions. The allele frequency distribution is expected to be approximately L-shaped under mutation-drift equilibrium, while a shift in this distribution is indicative of a recent bottleneck [42]. Although the program provides results under three possible mutation models; the Infinite Allele Model (IAM), the Stepwise Mutation Model (SMM) and the two-phase mutation model (TPM), we took into account only the TPM and the SMM model, which perform better for microsatellites datasets [43–45]. Simulation of heterozygosity at mutation-drift equilibrium distributions for the TPM model assumed 70% single-step mutations and 30% of multiple-step mutations. Significance was assessed using Wilcoxon’s signed rank test, as recommended for less than 20 markers.

The bottleneck analysis can only detect extreme reductions in population sizes that have occurred during the last 0.2–4.0 N_e_ generations [46]. Based on the average N_e_ estimations for *Ae. aegypti* populations worldwide [26, 47, 48] the program cannot detect bottlenecks that occurred more than ~1200 generations ago.

### Phylogenetic analysis

To infer the evolutionary relationships among populations we used Maximum Likelihood (ML) analysis, as implemented in RAxML [49], using 1000 bootstraps and the GTR model of evolution along with the CAT model of rate heterogeneity. For the runs we used the string “ASC” to apply an ascertainment bias correction to the likelihood calculations, and the standard correction by Lewis [50] when only variant sites are included in the data set, as directed by the software’s manual. The phylogenetic analysis was performed using the SNP dataset and randomly choosing two individuals per population (Fig. 1). The final dataset consisted of 62 individuals (including two *Aedes mascarensis* individuals that served as outgroup and were genotyped for the same SNPs using the *Ae. aegypti* SNP-chip). Since a major assumption in the phylogenetic analyses is the neutrality of the loci under study, we identified outlier SNPs (q-values < 0.05) using the *pcadapt* package [51] in R, which is based on Principal Components Analysis (PCA) and it does not require grouping individuals into populations. The SNPs identified as outliers were then filtered out to exclude SNPs that might be under selection. In total, 134 SNPs were excluded from the phylogenetic analysis as possibly being under selection. The final dataset after filtering to include only variant sites for this specific pruned dataset of 62 samples resulted in 19,092 SNPs that were used for the ML analysis.

### Inferring population history

Bayesian computation methods (ABC) [52] as implemented by DIYABC v.2.0.4 [53] were used to infer the population history of *Ae. aegypti* in the Black Sea. The ABC analysis was performed on the microsatellite dataset. We tested five scenarios to identify the origin of the Black Sea populations (Scenario 1: Black Sea originated from an admixed event between New World and Asia; Scenarios 2 and 5: New World origin; Scenario 3: Asian origin; and Scenario 4: African origin).

The best-fit scenario and confidence on the model of choice were evaluated using the DIYABC [53]. Divergence times were estimated in generations and the priors setting (for details see Additional file 1: Table S2) was based on the historical record information from previous studies [1, 7, 23, 26]. Given that the number of generations per year for *Ae. aegypti* is affected by the climatic conditions [54, 55], we collected climatic data (www.worldclim.com) for our Black Sea sampling localities and compared them with tropical and subtropical regions where *Aaa* is established (see Additional file 1: Table S3). The transformation of divergence time from generations to years for Black Sea region is challenging since studies estimating the number of generations per year have been mainly conducted for tropical populations and it is usually assumed an average of 10 generations per year (for details see [23, 55, 56]). However, considering that at least one quarter of the year the mean temperature in the Black Sea region is much lower than in other localities that established *Aaa* populations occurred (see Additional file 1: Table S3), it is possible that in Black Sea less than 10 generations per year exist. Thus, for the time estimates regarding the Black Sea region we provide a range assuming 6–8 generations per year. A mutation rate ranging from 9 × 10^-6^ to 1 × 10^-5^ was used based on rates reported in the literature for other Diptera species [57, 58]. Details on the effective population size and split time between regions used as priors for the ABC analysis are provided in Additional file 1: Table S2.

We did not run a DiyABC analysis on the SNP dataset due to the possible SNP ascertainment bias introduced during the SNP-chip design (the chip was designed to capture representative variation across different regions of the world [16]) that could affect the inference of demographic history through its effect on the Allele Frequency Spectrum-AFS [59–62]. Alternatively, we used the output of the ML analysis (see phylogenetic analysis section) to perform a Bayesian Binary MCMC (BBM) analysis on RASP [63] to reconstruct the ancestral area distribution of the Black Sea populations. For the MCMC analysis we ran 10 chains of 50,000 cycles, we set the maximum number of areas to four and we did not take the distribution area of outgroup into account for the analysis.

## Results

### Genetic diversity and differentiation

Sixty-five F_is_ values out of 685 (9.49%) population-by-locus comparisons deviated significantly from Hardy-Weinberg equilibrium (HWE) after sequential Bonferroni correction (α = 0.05), a level common for microsatellites and most often due to rare null alleles [14, 23].

**Fig. 2:**
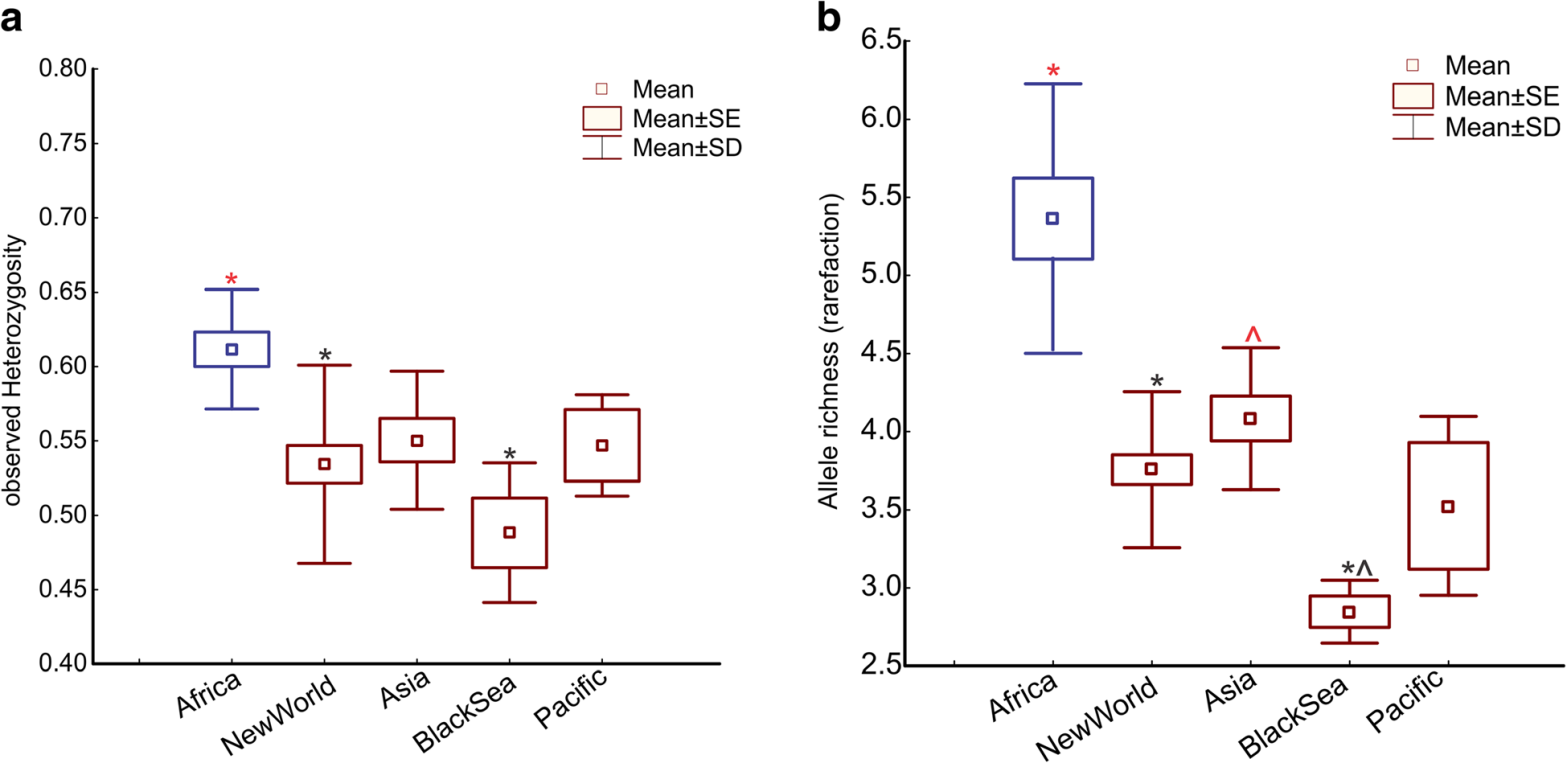
Genetic diversity estimates based on the microsatellite dataset. Observed heterozygosity (**a**) and number of alleles per populations using rarefaction (**b**). Populations are grouped by region and the non-parametric Kruskal-Wallis test was implemented to test for differences between groups (*P* < 0.05). The groups that differ significantly are indicated with matching symbols of different colors (asterisk or arrow tip) above the respective boxplot. The mean, standard deviation (SD) and the standard error (SE) are presented in each panel

Population genetic statistics for each population for the microsatellite dataset are provided in Additional file 1: Table S4. Populations in the range of *Aaa* that includes the Black Sea samples have significantly lower levels of allelic richness (AR) and observed heterozygosity (Ho) compared with *Aaf* (Fig. 2; Kruskal-Wallis H-test: *χ*^2^ = 16.522, *df* = 4, *P* = 0.0024 and *χ*^2^ = 14.035, *df* = 4, *P* = 0.0072). Focusing only on *Aaa*, the Black Sea region shows similar levels of Ho when compared with the other regions, but lower levels of AR compared to Asia (Fig. 2). Private allelic richness (Np) for the full dataset (56 populations) was low (Np ≤ 0.09) in all cases with the exception of some African and Argentinean populations. Analysis of the Black Sea populations’ dataset (only Turkey and Georgia) revealed that the Np richness range between 0.09–0.17, but no private alleles were found in the Black Sea populations, relative to the populations of the New World or Asia. However, if we consider the region level instead of the population level, Black Sea has, on average, a small proportion of private alleles (Africa 2.36, New World 0.50, Asia 0.64, Pacific 0.11 and Black Sea 0.13).

**Fig. 3:**
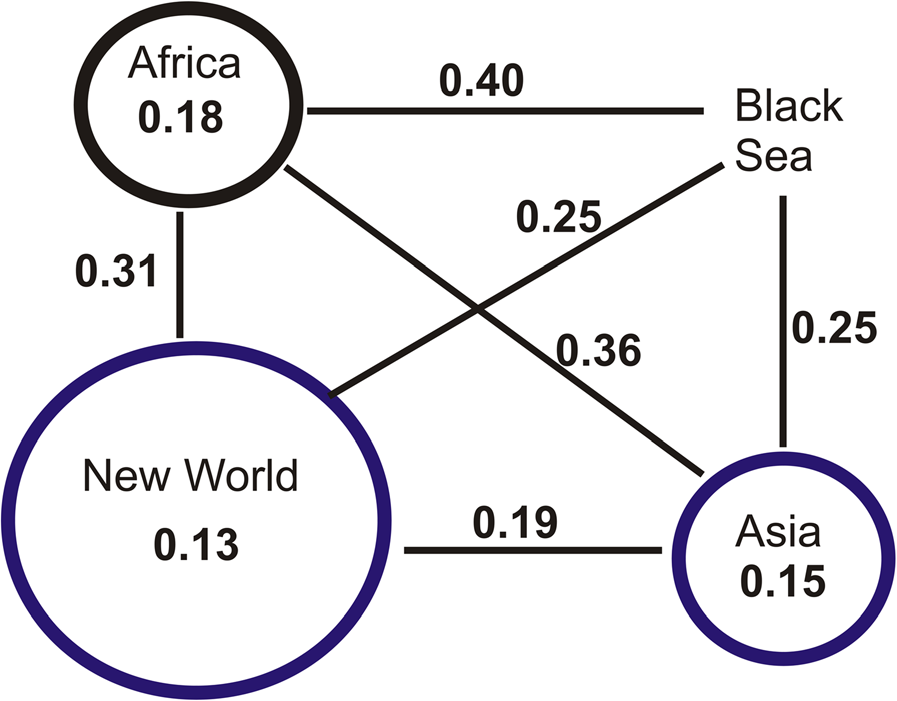
Genetic differentiation. Average pairwise *F*_ST_ values as estimated based on the SNP dataset, between and within regions. Numbers in circles are the mean *F*_ST_ among samples of that group. Mean *F*_ST_ values between groups are indicated next to the lines connecting them. Sizes of the circles are proportional to the number of populations sampled. Colors are consistent with Fig. 1

The mean genetic differentiation between the geographical regions, *F*_ST_ based on SNP chip data are summarized in Fig. 3. All pairwise *F*_ST_ estimations using the SNP dataset were significant (*P* < 0.05, using 1000 permutations). This figure is comparable to Fig. 2b presented in [24] that uses RAD sequencing data, providing evidence that the patterns are robust and not dependent on type of genetic data analyzed.

### Genetic structure

**Fig. 4:**
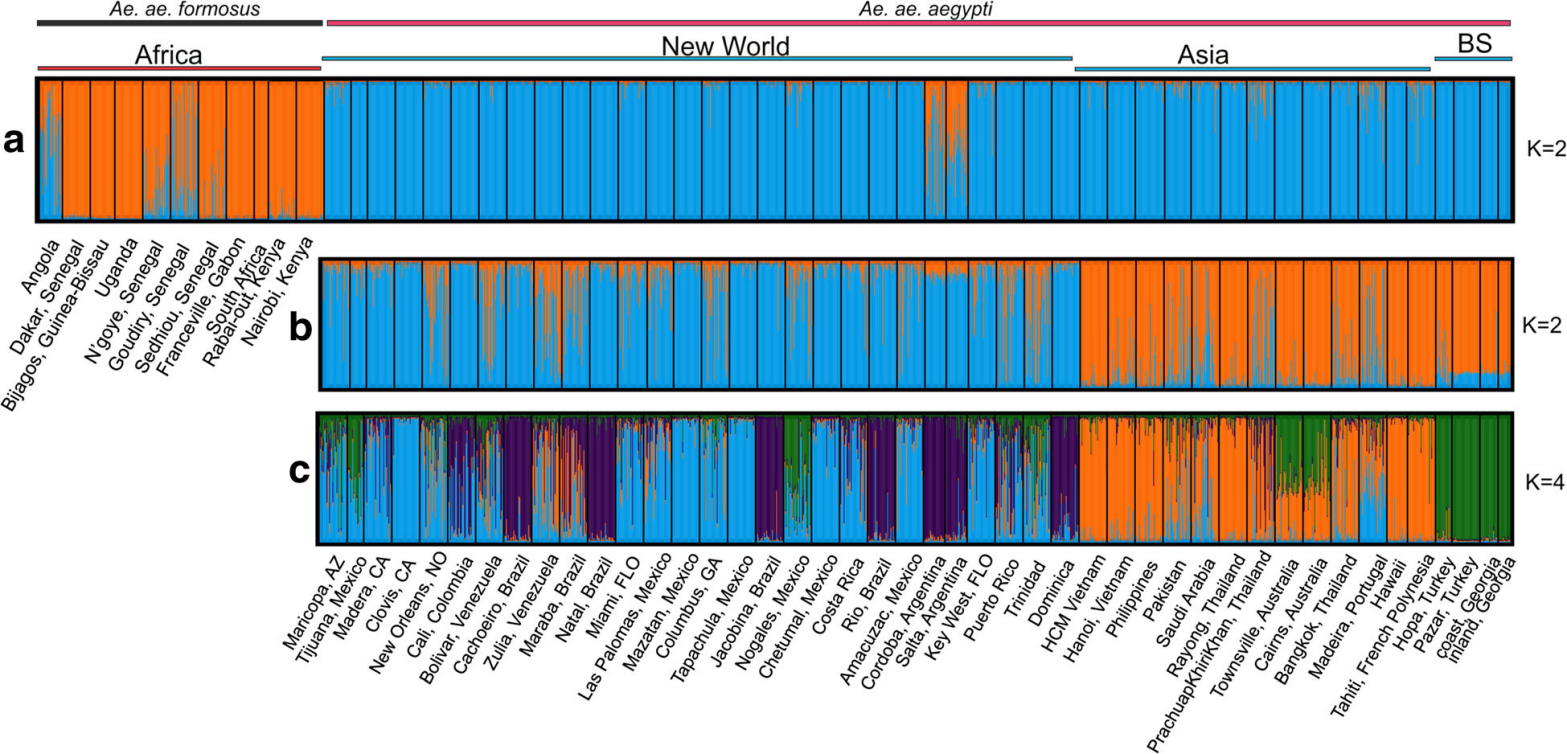
STRUCTURE bar plots for all *Ae. aegypti* populations in the microsatellite dataset. Two genetic clusters were supported using a representative set of populations from the entire distribution (**a**), while two (**b**) and four (**c**) clusters were supported focusing on the populations outside Africa. Population names are reported on the X axis. The Y axis reports the probability of each individual (Q-value) assigned to one of the genetic groups identified by STRUCTURE, which are represented by different colors. Each bar represents an individual. Individuals with 100% assignment to one group are identified by a single color. Individuals with mixed ancestry are represented by bars with different percentages of colors. The thick black lines within the plots indicate population limits. The bars on the top apply to all three plots. *Abbreviation*: BS, Black Sea

**Fig. 5:**
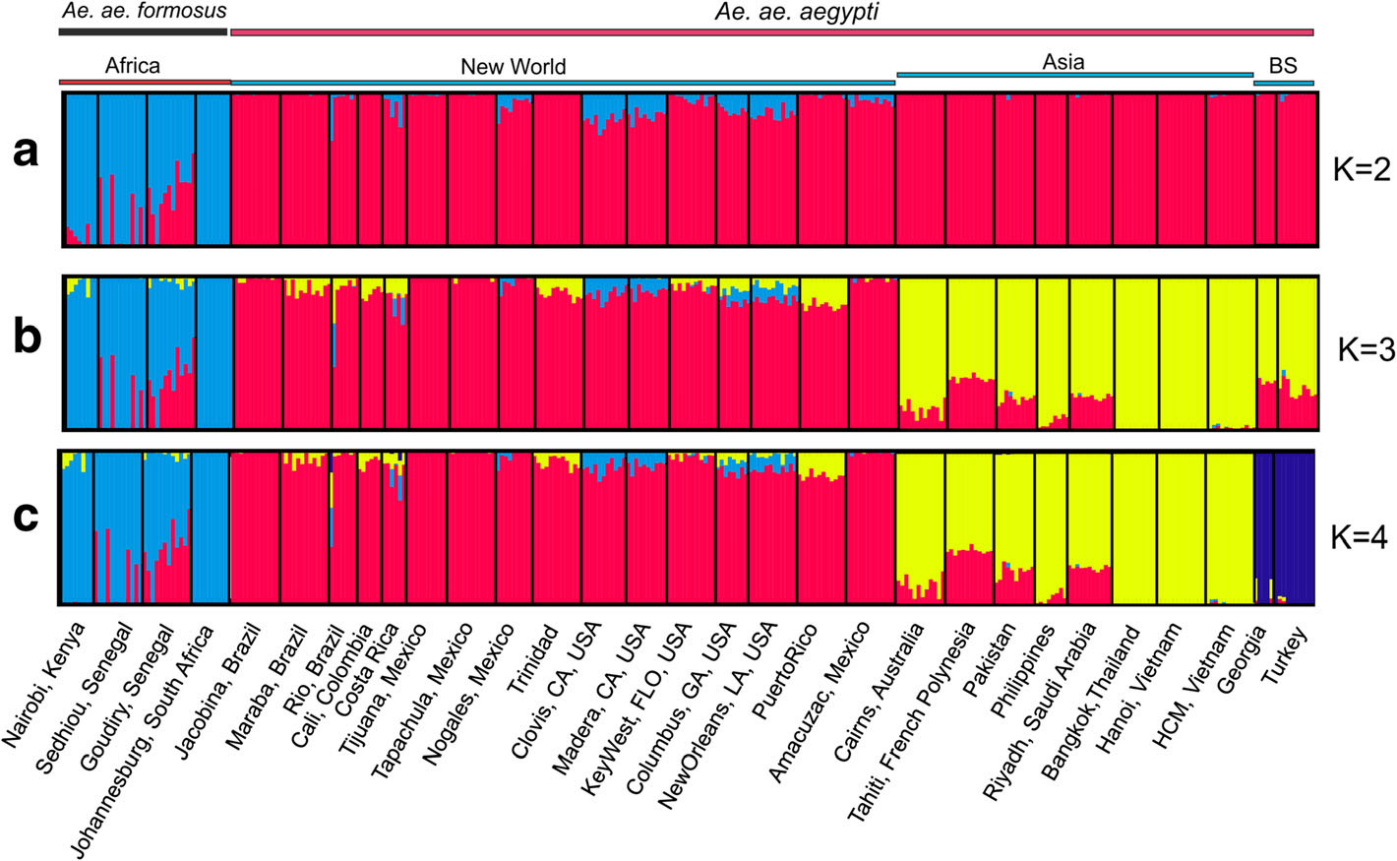
STRUCTURE bar plots for all *Ae. aegypti* populations present in the SNP dataset as plotted for two (**a**), three (**b**) and four (**c**) genetic clusters. Population names are reported on the X axis. For details see legend of Fig. 4

Structure analyses on both datasets showed that the Black Sea populations are genetically distinct (Evanno method; *K* = 2) from sub-Saharan *Aaf*, clustering within *Aaa* populations (Figs. 4a, 5a). Subsequently, within *Aaa*, Black Sea clusters first with Asia (Figs. 4b, 5b) and then as a distinct group (Figs. 4c, 5c). Structure analyses on the smaller datasets, normalizing the number of populations per region with those of the Black Sea region, confirmed that the Black Sea is of the *Aaa* type (Additional file 1: Figure S1) and according to the Evanno et al. method [30], forms a distinct group within this range (Additional file 1: Figure S2).

**Fig. 6:**
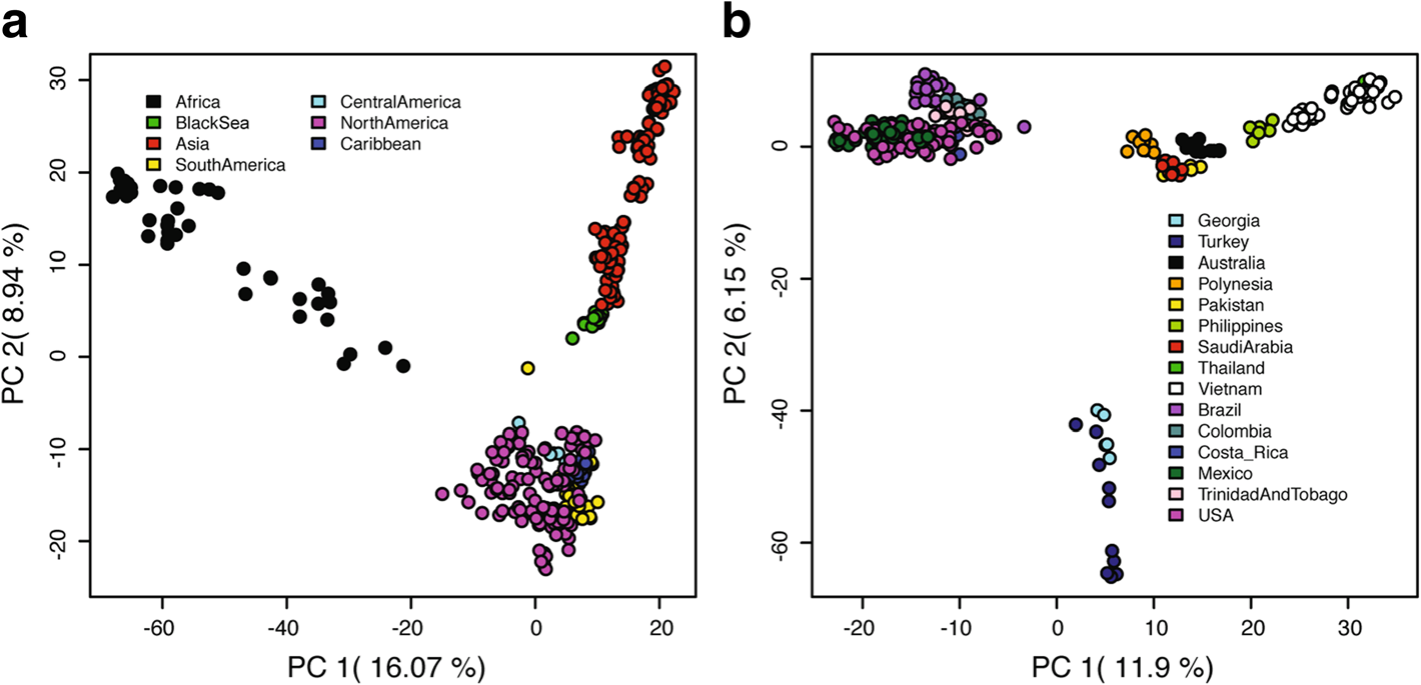
Principal Components Analysis (PCA). PCA was performed on the global (**a**) and the *Ae. ae. aegypti* (**b**) SNP dataset as implemented and plotted in the *LEA* package, presenting the projection of all individual mosquitoes on the first two PCs. Populations originated from different regions are presented with different colors, as shown in the respective insets

PCA analysis on both datasets also confirmed the distinction between *Aaf* and *Aaa* populations (Fig. 6a, Additional file 1: Figure S3a). Focusing on *Aaa*, Black Sea samples again form a group distinct from both Asia and New World (Fig. 6b, Additional file 1: Figure S3b).

DAPC on the worldwide microsatellite and SNP chip datasets indicated (by the Bayesian Information Criterion-BIC) 20 DAPC-groups, with the majority (microsatellites dataset; 68 out of the 91) or all (SNP chip dataset) of the Black Sea individuals forming a single distinct group (Additional file 1: Figure S4). Focusing on the Black Sea, BIC supported three groups, (Additional file 1: Figure S5) with no clear distinction between Georgia and Turkey populations.

### Assignment test

Self-assignment tests on the microsatellite dataset correctly assigned 80.9% individuals to their population of origin and 99.9% were correctly assigned to their original continent (America, Asia). Self-assignment tests on the SNP dataset correctly assigned 97.7% of the individuals to their original population with mis-assignments usually occurring within the same country, with the exception of Costa Rica, which was usually mis-assigned to Florida.

When using the worldwide reference panel, individuals from the Black Sea were assigned primarily to North American populations, with scores of 50% or higher, based on microsatellite data (Additional file 1: Figure S6). A large number of individuals from the Turkish populations were assigned with the highest average assignment probability to New Orleans (America), closely followed by Ho Chi Minh (Asia) and Miami (America). New Orleans and Miami were also assigned a large number of individuals from the coast population in Georgia, based on highest average assignment probability, while individuals from the inland-Georgia population were assigned with high probabilities to Pakistan, Thailand, Brazil and Mexico (Additional file 1: Figure S6).

For the SNP dataset, we ran the analysis multiple times in panels of 4000 SNPs. The Black Sea individuals were assigned to Mexico, Vietnam, New Orleans, Puerto Rico, and Pakistan, with frequencies as shown in Additional file 1: Figure S6. The majority of individuals were assigned with scores of 70% or higher to Puerto Rico (Caribbean), followed by Ho Chi Minh (Asia).

### Bottleneck effect

Bottleneck analysis on the Black Sea samples provided evidence of a recent bottleneck in all four populations. Specifically, both tests (Wilcoxon and mode shift) and both models (TPM, SMM) showed significant (Wilcoxon test one tail for H excess *P* = 0.002 and *P* = 0.046) demographic changes for the Turkey-Hopa population, while for the other three populations (Turkey-Pazar, Georgia-inland, Georgia-coast) only Wilcoxon test and only the TPM model showed marginally significant (Wilcoxon test one tail for H excess 0.04 < *P* < 0.05) signs of bottleneck.

### Phylogenetic analysis

**Fig. 7:**
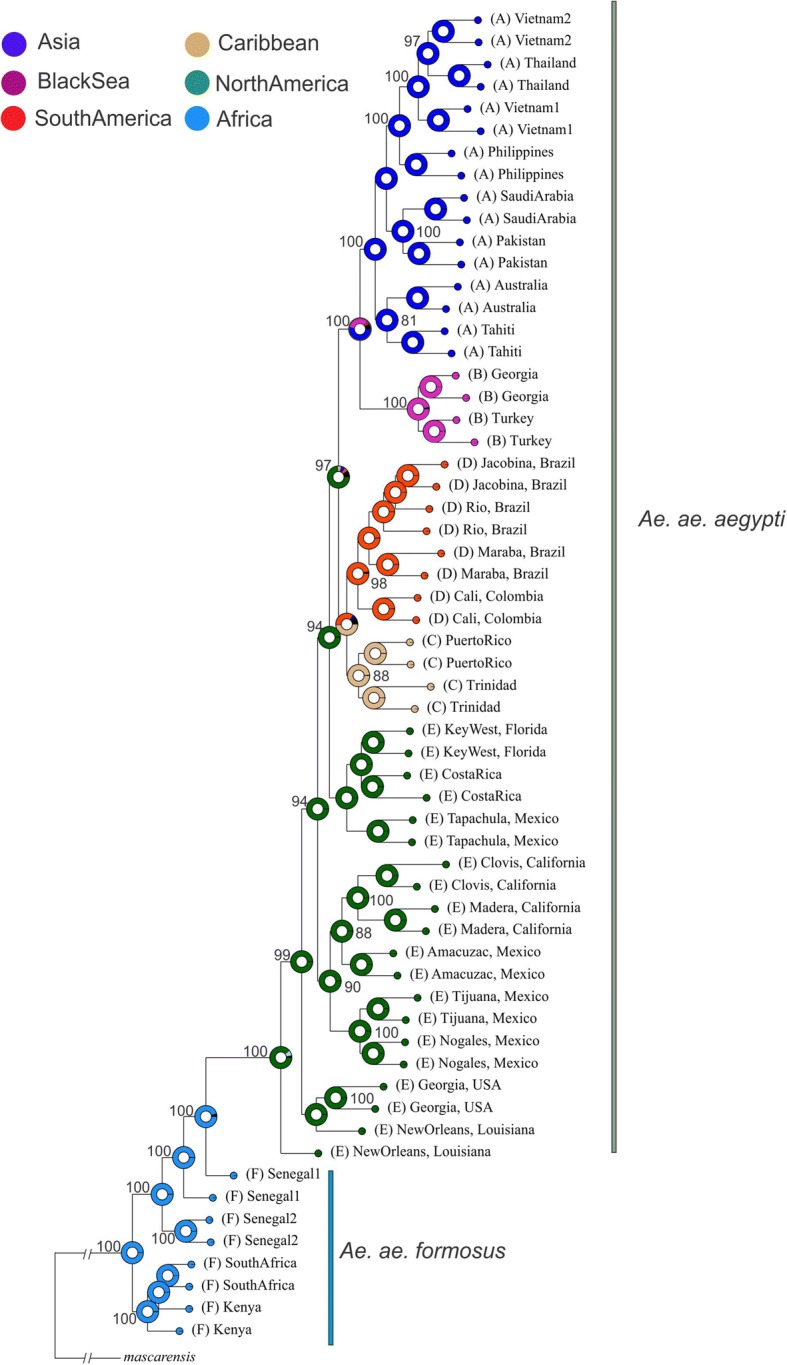
Maximum Likelihood (ML) rooted phylogenetic tree reconstructed using ~19,000 SNPs. *Aedes mascarensis* was used as outgroup. Bootstraps are presented on the nodes, and for visualization purposes values < 88 are not shown. This ML tree was used for Bayesian Binary MCMC (BBM) analysis. For BBM, six geographical regions were pre-defined based on the current distribution of the sampled populations and they are shown with different tip colors. The colors in the pies on the nodes indicate the probabilities of ancestry of the individuals that were included in the respective clade

The rooted ML phylogenetic tree constructed from the SNP dataset is presented in Fig. 7. All *Aaa* populations form a monophyletic group distinct from all *Aaf* populations. Within *Aaa*, Black Sea populations form a monophyletic group that is basal to the Asian clade and the sister clade to the South America-Caribbean clade.

### Inferring population history

**Fig. 8:**
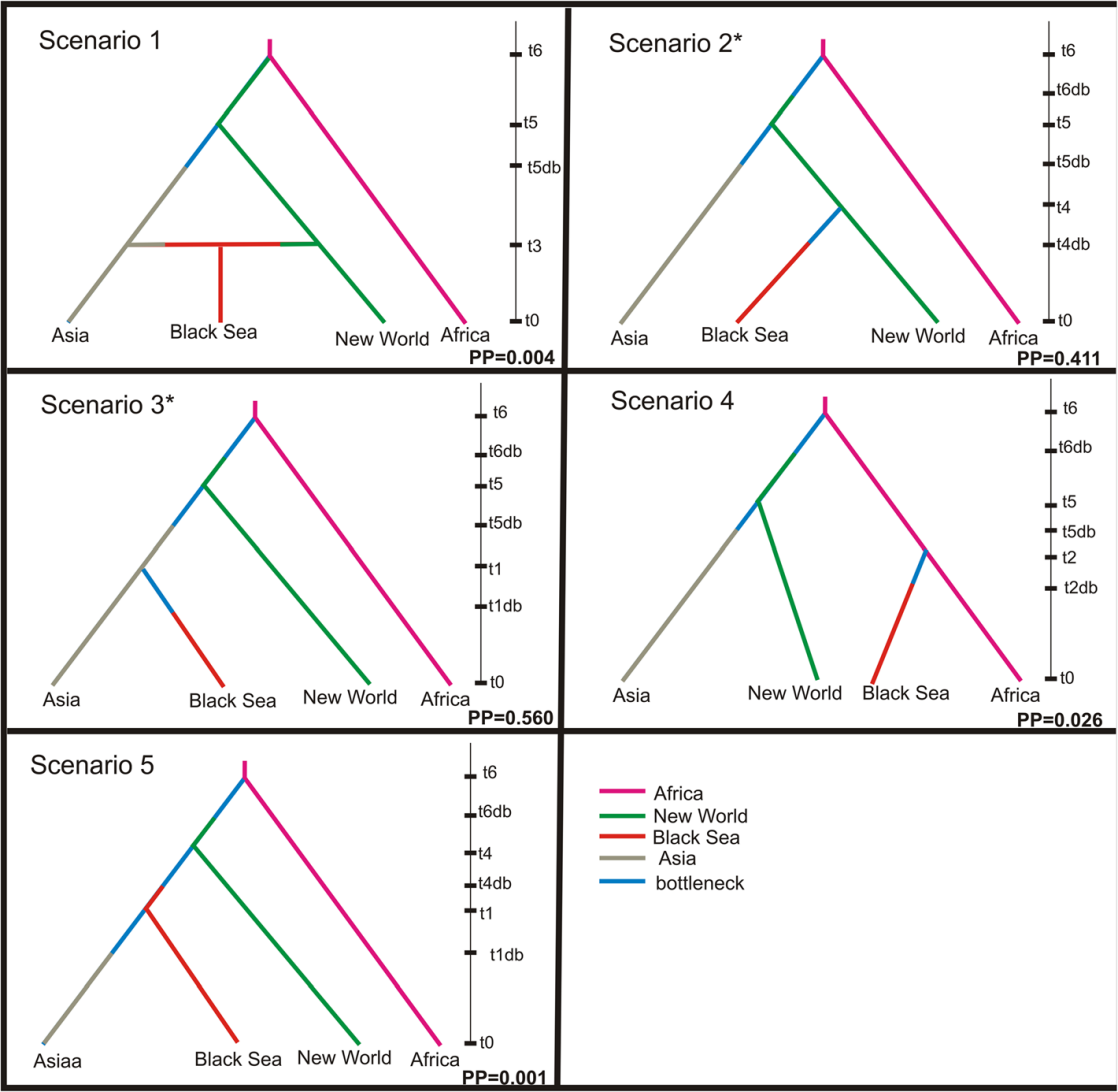
Evaluated evolutionary scenarios of *Aedes aegypti* invasion to the Black Sea region, using Approximate Bayesian Computation (ABC) inference, as implemented by the DIYABC software. The time scale on the right of each scenario is the relative time, with t0 representing the present and increasing values going back in time. Numerical estimates of the generations associated with these times are in Additional file 1: Table S2. Posterior probabilities (PP) are shown for each scenario. The two best supported scenarios are indicated by asterisk

Figure 8 shows the scenarios tested for the diaspora of *Ae. aegypti* from Africa. The analysis indicated two scenarios with the highest Posterior Probability (PP); Scenario 3 (PP = 0.56) supporting that the species arrived in New World from Africa ~500 years ago, then from the New World invaded Asia ~150 years ago and later the Black Sea from Asia ~150–110 years ago (assuming 6–8 generations per year); and Scenario 2 (PP = 0.41) indicating that Asia and Black Sea may have been two independent invasions from the New World. Detailed information on the results of the demographic analysis is provided in Additional file 1: Table S2.

As mentioned above, Asia-Black Sea and South America-Caribbean are sister clades. RASP analysis (Fig. 7) supported that the ancestral area of these two major clades is likely North America (probability 0.74). However, focusing on the ancestral area of the Asia-Black Sea region, there is a degree of uncertainty. The probability of Asia being the ancestral area is 0.53, compared to 0.34 for the Black Sea ancestral [the remaining 0.13 is either to be unknown (black proportion in the pie in Fig. 7) or a continuous region (brown proportion in the pie) covering both Black Sea and Asia]. Thus the most likely scenario according to RASP (probability of 0.51), suggests that mosquitoes arrived in Asia from New World and then went to the Black Sea region through dispersal from Asia.

## Discussion

Our population genetics analyses on the Black Sea populations using two types of genetic markers (12 microsatellites and ~19,000 SNPs) revealed relatively low differentiation between the populations within this region and subtle genetic structure (Additional file 1: Figure S5), which is expected considering the intense road traffic between Georgia and Turkey. Thus, we consider the *Ae. aegypti* populations of Turkey and Georgia as parts of a single Black Sea genetic unit that we will refer to as BS. Placing this genetic unit into the worldwide reference panel of *Ae. aegypti* populations, all analyses concluded that BS populations clearly belong to the subspecies *Aaa* and are distantly related to African *Aaf*. Within *Aaa*, BS, Asia and New World are all equally genetically differentiated from each other, *F*_ST_ ~0.19–0.25 (Fig. 3).

The high levels of genetic differentiation (see Figs. 3, 4c, 5c, 6b, Additional file 1: Figures S2-S4) combined with the fact that BS was first reported in 2008 [11] raise questions on the origin(s) and the approximate age of BS. It is well documented that *Ae. aegypti*, although present in Europe during 1800s and 1900s, has been rarely recorded since the 1950s (see [7] and references therein) due to a combination of factors (e.g. insecticides, improvements in sanitation). However, in recent years the species re-appeared in the periphery of Europe, with established populations reported in Madeira in 2004 [64] and in the Black Sea region (Russia and Georgia) in 2001 [11]. The Madeira population is very likely a recent re-invasion, as suggested by previous studies [10, 65]. The Madeira population shares mitochondrial alleles with South American populations [65], clusters together with South America and Asia in the microsatellites structure analysis [23], and falls within the South America-Caribbean and Asia clouds in our DAPC and PCA analyses (see Additional file 1: Figures S3 and S4). In contrast, BS is quite different in being equally and strongly genetically differentiated from both Asia and the New World (Fig. 3).

If the Black Sea population is a recent invasion [occurring in 2001 (first record in Sochi area) or shortly before] it should have close genetic affinities with some other population(s) in the reference panel, as observed in several other cases of recent re-invasions around world [65–67]. As noted in the Results section, individual assignment tests of random individuals in our data set are re-assigned to their correct population of origin > 98% of the time with SNP chip data. While BS shows genetic affinity with Asia (Figs. 4b, 5b, 7), this relationship is in the context of broader biogeographical patterns and the evidence is not consistent with a recent invasion that occurred during the last 10–15 years from Asia (Figs. 3, 6b, Additional file 1: Figures S2-S4). On the contrary, the high genetic differentiation combined with assignment tests, and estimated divergence times (Additional file 1: Table S2; on average ~950 generations back) support the hypothesis that the Black Sea is a remnant population that has existed in the region despite the apparent elimination of this species in the Mediterranean, and that remained cryptic until its detection in 2001. Bottleneck signatures found in this population are also consistent with the remnant hypothesis. Importantly, during the years after presumed eradication, *Ae. aegypti* was sporadically reported in Turkey (in 1961, 1984, 1992, 1993 and 2001) [7], suggesting that small populations of the species were maintained there after the 1950s, when it was reported eliminated from the Mediterranean. The relatively high genetic diversity (Fig. 2) in the Black Sea is consistent with this being an older population. However, the direct invasion source of the BS population is difficult to be found because (a) the gene flow between BS and modern Asian-North American populations may obscure the true pattern and (b) the population of origin could be a population not in our reference dataset (e.g. old Mediterranean populations or Russian population).

The most likely phylogeographical scenario, supported by two independent analyses (ABC and RASP) is that *Ae. aegypti* from the New World arrived in Asia ~150 years ago, coinciding with previous studies and the historic record [1, 23]. However, it is unclear if the species invaded the Black Sea region from Asia (Scenario 3; Fig. 8, Structure plots in Figs. 4, 5) or BS originated directly from the New World (Scenario 2; Fig. 8 and assignment tests) since both ABC and RASP analyses retain a degree of uncertainty regarding the relationship of New World-Black Sea-Asia (see Figs. 7, 8). Specifically, in ABC analysis both scenarios (Scenario 2 and 3) are almost equally probable and in the RASP analysis there is a probability of 0.3 (compared with 0.5) for BS being ancestral to all Asian populations. Inferring the exact biogeographical relationships between these regions is challenging for two reasons: (i) the lack of the now extinct Mediterranean populations from our analyses in order to test if BS is a remnant of this population; and (ii) the difficulty in estimating the actual age of this population. As mentioned above (see Methods section) the transformation from generations to years depends on the number of generations per year, but we lack such estimates for the BS region or regions with similar climatic conditions. However, the BS is considerably cooler than tropical Africa and Americas, so the number of generations/year must be lower. This, combined with the fact that the estimated divergence time (in generations) indicated by the analysis is relatively broad (see Additional file 1: Table S2), prevents us from precise estimates. Here, we chose to present the mean time divergence estimates for BS as we did for all the *Ae. aegypti* populations studied, however, we note that given the limitations presented above, we cannot reject that the actual age of the BS population could exceed the ~100–150 years, which would allow the estimate to be consistent with historical epidemiological data.

Given the appearance of yellow fever in the New World no later than the 17th Century and perhaps as early as the 15th [68], the estimated time of founding of the New World is about 500 years ago (see also Additional file 1: Table S2), shortly after the Europeans arrived in the New World. The first appearance of established yellow fever and dengue in Europe were in Spain in 1801–1804 (while cases of yellow fever are reported in European ports prior to this time, annually repeated epidemics are documented for first time in early 1800s, indicating establishment of year-round breeding *Ae. aegypti*). Urban dengue and chikungunya first appeared in Asia in 1879–1890. The opening of the Suez Canal in 1869 has been proposed as the path that facilitated this dispersal [4]. The historic epidemiological record and the estimated time of *Ae. aegypti* arrival to Asia ~150 years ago reinforce this hypothesis. Although we included a similar scenario in our ABC analysis (Fig. 8; Scenario 5), the absence of data on Mediterranean populations prior to 1950 likely accounts for the lack of support for the hypothesis.

## Conclusions

The use of two types of markers and several population genetics analyses revealed high genetic differentiation between the Black Sea population and an extensive reference panel of *Ae. aegypti* populations distributed worldwide. This high genetic differentiation combined with the mean approximate age estimated for this population of > 100 years, signs of a bottleneck, and the sporadic reports regarding the presence of the species in Turkey after 1950s, support the hypothesis that the present populations in the Black Sea are recently expanded populations of small remnants that existed in the area long before its discovery in 2008. We could not definitively determine whether the Black Sea population is a remnant of old Mediterranean populations that has been hypothesized to be the invasion source of Asian populations, or is an independent introduction from Asia.

## Additional file

Additional file 1: **Table S1.** Population information for the *Ae. aegypti* samples used in this study. Populations represented in both microsatellite and SNP datasets are indicated by bold characters. **Table S2.** Priors and posteriors for the ABC analysis testing scenarios on the origin of Black Sea populations. **Table S3.** Climatic data for Black Sea sampling localities (indicated by bold characters) and indicative worldwide sampling localities where *Ae. aegypti* is established as downloaded from www.worldclim.com. **Table S4.** Genetic diversity. Summary of the population genetic diversity statistics for the 56 populations of *Ae. aegypti* consisting the microsatellite dataset. **Figure S1.** STRUCTURE bar plots based on the microsatellite dataset, including equal number of populations from each region. Population names are reported on their X axes. For each STRUCTURE run only the number of genetic clusters supported by the Evanno method is presented. For details see legend of Fig. 4. **Figure S2.** STRUCTURE bar plots based on the microsatellite dataset, including equal number of *Ae. ae. aegypti* populations from each region. For each STRUCTURE run only the number of genetic clusters supported by the Evanno method is presented. For details see legend of Fig. 4. **Figure S3.** Principal Components Analysis (PCA) on the global (**a**) and the *Ae. ae. aegypti* (**b**) microsatellite dataset as implemented and plotted using the ade4 package in R. **Figure S4.** Discriminant Analysis of Principal Components (DAPC) for the *Ae. ae. aegypti* populations based on the microsatellite dataset. **Figure S5.** Discriminant Analysis of Principal Components (DAPC) for the *Ae. aegypti* populations collected from Turkey and Georgia based on the microsatellite dataset. **Figure S6.** Assignment test as implemented in Geneclass2 for the Black Sea populations and using the remaining worldwide populations as reference panel. (PDF 8863 kb)

## Abbreviations

*Aaa*: *Aedes aegypti aegypti*
*Aaf*: *Aedes aegypti formosus*
ABC: Approximate Bayesian Computation methods
AFS: Allele Frequency Spectrum
AR: Allelic richness
BBM: Binary Bayesian MCMC method
BIC: Bayesian Information Criterion
BS: Black Sea
DAPC: Discriminant Analysis of Principal Components
Fis: Inbreeding coefficient
*F*_ST_: Fixation index
GSM: Generalized Stepwise Mutation Model
GTR: General Time Reversible
He: Expected heterozygosity
Ho: Observed heterozygosity
HWE: Hardy-Weinberg equilibrium
IAM: Infinite Allele Model
MCMC: Markov chain Monte Carlo
ML: Maximum Likelihood
Ne: Effective population size
Np: Private allelic richness
PCA: Principal Components Analysis
RASP: Reconstruct Ancestral State in Phylogenies
RAxML: Randomized Axelerated Maximum Likelihood
SD: Standard deviation
SE: Standard error
SMM: Stepwise Mutation Model
SNPs: Single nucleotide polymorphisms
TPM: Two-phase Mutation Model
