# Supplementary material for: Aedes aegypti in the Black Sea: recent introduction or ancient remnant?
Source: Parasit Vectors. 2018 Jul 6;11:396. doi: 10.1186/s13071-018-2933-2 (PMC6035450; doi:10.1186/s13071-018-2933-2)
Supplement: Supplementary file 1 — Table S1. Population information for the Ae. aegypti samples used in this study. Populations represented in both microsatellite and SNP datasets are indicated by bold characters. Table S2. Priors and posteriors for the ABC analysis testing scenarios on the origin of Black Sea populations. Table S3. Climatic data for Black Sea sampling localities (indicated by bold characters) and indicative worldwide sampling localities where Ae. aegypti is established as downloaded from www.worldclim.com. Table S4. Genetic diversity. Summary of the population genetic diversity statistics for the 56 populations of Ae. aegypti consisting the microsatellite dataset. Figure S1. STRUCTURE bar plots based on the microsatellite dataset, including equal number of populations from each region. Population names are reported on their X axes. For each STRUCTURE run only the number of genetic clusters supported by the Evanno method is presented. For details see legend of Fig. 4. Figure S2. STRUCTURE bar plots based on the microsatellite dataset, including equal number of Ae. ae. aegypti populations from each region. For each STRUCTURE run only the number of genetic clusters supported by the Evanno method is presented. For details see legend of Fig. 4. Figure S3. Principal Components Analysis (PCA) on the global (a) and the Ae. ae. aegypti (b) microsatellite dataset as implemented and plotted using the ade4 package in R. Figure S4. Discriminant Analysis of Principal Components (DAPC) for the Ae. ae. aegypti populations based on the microsatellite dataset. Figure S5. Discriminant Analysis of Principal Components (DAPC) for the Ae. aegypti populations collected from Turkey and Georgia based on the microsatellite dataset. Figure S6. Assignment test as implemented in Geneclass2 for the Black Sea populations and using the remaining worldwide populations as reference panel. (PDF 8863 kb) [file 13071_2018_2933_MOESM1_ESM.pdf]

Additional File 1

Table S1. Population information for the *Ae. aegypti* samples used in this study. Populations represented in both microsatellite and SNPs datasets are indicated by bold characters. In the analyses focused on the broader geographical region instead of the population level the populations were grouped as indicated inhe "Broad Region" column.

Population	Country	Broad Region	Collected	N (msats/SNPs)
Francesville & Lope forest	Gabon	Africa	2014	34/0
Bijagos	Gouinea-Bissau	Africa	2009	30/0
Rabai-out	Kenya	Africa	2009-2012	34/0
**Nairobi**	Kenya	Africa	2012	34/8
Dakar	Senegal	Africa	2005	34/0
N'goye	Senegal	Africa	2007	34/0
**Goudiry**	Senegal	Africa	2012	34/12
**Sedhiou**	Senegal	Africa	2012	34/12
Kichwamba	Uganda	Africa	2009	34/0
**Johannesburg**	South Africa	Africa	2015	18/9
Luanda	Angola	Africa	2016	28/0
Townsville	Australia	Asia	2009	34/0
**Cairns**	Australia	Asia	2013	34/12
**Tahiti**	French Polynesia	Pacific	2010	34/12
**Attock**	Pakistan	Asia	2014	34/10
**Cebu City**	Philippines	Asia	2013	34/8
**Riyadh**	Saudi Arabia	Asia	2012	34/11
Rayong	Thailand	Asia	2009	34/0
Prachuabkhirikan	Thailand	Asia	2009	34/0
**Bangkok**	Thailand	Asia	2013	34/11
**Ho Chih Minh**	Vietnam	Asia	2013	34/12
**Hanoi**	Vietnam	Asia	2013	34/12
Zugdidi (coast)	Georgia	Black Sea	2015	22/0
**Tbilisi (inland)**	Georgia	Black Sea	2015	15/5
**Hopa**	Turkey	Black Sea	2015	20/10
Pazar	Turkey	Black Sea	2015	34/0
Cordoba	Argentina	New World	2006	26/0
Salta	Argentina	New World	2003, 2014	27/0
Cachoeiro	Brazil	New World	2008	34/0
**Maraba**	Brazil	New World	2010	34/12
Natal	Brazil	New World	2010	34/0
**Jacobina**	Brazil	New World	2013	34/12
**Rio de Janeiro**	Brazil	New World	2014	34/7
**Cali**	Colombia	New World	2013	34/6
Dominica	Commonwealth of Dominica	New World	2009	34/0
**Siquirres**	Costa Rica	New World	2014	34/6
**Tijuana**	Mexico	New World	2013	20/10
Las Palomas, GRO	Mexico	New World	2012	34/0
Mazatan, CHP	Mexico	New World	2012	34/0

**Tapachula Norte, CHP**	Mexico	New World	2012	34/12
**Nogales, SON**	Mexico	New World	2013	34/9
Chetumal, QRO	Mexico	New World	2013	34/0
**Amacuzac, MOR**	Mexico	New World	2014	34/12
Madeira	Portugal	Atlantic	2012	34/0
**Patillas**	Puerto Rico	New World	2014	34/12
**Trinidad**	Republic of Trinidad and Tobaco	New World	2014	34/12
Maricopa County, AZ	USA	New World	2013	34/0
**Madera, CA**	USA	New World	2013	34/10
**Clovis, CA**	USA	New World	2013	34/10
**New Orleans, NO**	USA	New World	2012	34/12
Miami, FL	USA	New World	2011	34/0
**Columbus, GA**	USA	New World	2011	34/8
**North Key West, FL**	USA	New World	2013	34/12
Hawaii	USA	Pacific	2010	25/0
Bolivar	Venezuela	New World	2004	34/0
Zulia	Venezuela	New World	2004	34/0
Aedes mascarensis	Mauritius	Indian Ocean	2014	0/2*
**Total**				**1,795/308**

*used as outgroup only for the phylogenetic analysis

Table S2. Priors and posteriors for the ABC analysis testing scenarios on the origin of Black Sea populations. Priors for splitting time and for population size are all uniform distributions. Posterior probabilities for each scenario depicted in Fig. 2 are shown with 95% confidence intervals. Error and posterior distributions of parameters (mean with 90% confidence interval) are reported for the best-fit scenarios. Divergence times are measured in generations.

Parameter	Details	Prior	Posterior
Colonization scenario	Scenario 1 – Black Sea from merge between Asia and New World	N/A	0.004 [0.000, 0.023]
	**Scenario 2–** Black Sea from New World	N/A	**0.411 [0.391, 0.430]**
	**Scenario 3 –** Black Sea from Asia	N/A	**0.560 [0.545, 0.574]**
	Scenario 4 – Black Sea from Africa	N/A	0.026 [0.007, 0.044]
	Scenario 5 – Black Sea from New World	N/A	0.001 [0.0000, 0.019]
**SCENARIO 2**			
Effective population size	Africa	100 – 450,000	199,000 [82,900 – 370,000]
	New World	100 – 400,000	70,400 [14,400 – 207,000]*
	Black Sea	100 – 400,000	80,800 [55,300 – 232,000]*
	Asia	100 – 400,000	193,000[40,800 – 350,000]*
[1]Split time	New World from Africa	4,500 – 6,000	5,340 [4,720 – 5,930]
	Asia from America	500 – 1,800	1,550 [1,080 –1,790]
	Black Sea from America	100 – 1,800	876 [305 – 1,630]
Confidence	Type I error (Simulated under scenario 2)	N/A	0.120
	Type II error (Simulated under scenario 1)	N/A	0.000
	Type II error (Simulated under scenario 3)	N/A	0.180
	Type II error (Simulated under scenario 4)	N/A	0.012
	Type II error (Simulated under scenario 5)	N/A	0.032
**SCENARIO 3**			
Effective population size	Africa	100 – 450,000	207,000 [88,500 – 379,000]
	Asia	100 – 400,000	196,000 [41,800 – 351,000]*
	New World	100 – 400,000	72,700 [14,200 – 205,000]*
	Black Sea	100 – 400,000	79,900[55,000 –231,000]*
[1]Split time	New World from Africa	4,500 – 6,000	5,350 [4,730 – 5,930]
	Asia from America	500 – 1,800	1,570 [981 –1,750]
	Black Sea from Asia	100 – 1,800	953 [431 – 1,490]
Confidence	Type I error (Simulated under scenario 3)	N/A	0.300
	Type II error		

	(Simulated under scenario 1)	N/A	0.002
	Type II error (Simulated under scenario 2)	N/A	0.100
	Type II error (Simulated under scenario 4)	N/A	0.000
	Type II error (Simulated under scenario 5)	N/A	0.044
Mutation rate	Microsatellite –Generalized Stepwise Mutation Model (GSM)	9.0×10^{-06}-1×10^{-05}	9.5×10^{-06} [9.07×10^{-06} - 9.96×10^{-06}]

[1] Time in generations.

* Cases where the median of the absolute error/true value is > 0.3 that indicate low accuracy in the estimation of the specific parameter and scenario.

Table S3. Climatic data for Black Sea sampling localities (indicated by bold characters) and indicative worldwide sampling localities where *Ae. aegypti* is established as downloaded from www.worldclim.com.

Site	TempAnnual	maxTempWarmMonth	minTempColdMonth	meanTempWetQuar	meanTempDryQuar	meanTempWarmQuar	meanTempColdQuar
Nairobi	17.8	26.3	10.1	18.7	16.2	19.1	15.9
Johannesburg	16.2	26.5	2.2	19.9	10.8	20.2	10.7
Bangkok	28	34.6	20.3	28.1	26.2	29.5	26
Hanoi	23.9	33.1	13.4	28.5	17.5	29.1	17.5
**Zugdidi**	**13.6**	**27.6**	**0.4**	**21.8**	**12.2**	**21.8**	**5.2**
**Tsereteli**	**11.8**	**29.2**	**-4.1**	**19.7**	**1.3**	**21.9**	**1.3**
**Hopa**	**12.4**	**25.4**	**0.5**	**10.1**	**10.5**	**19.9**	**4.9**
**Pazar**	**8.1**	**23.3**	**-5.5**	**11.8**	**0.3**	**16.8**	**-1.2**
Jacobina	23	30.3	15.7	24.5	22.5	24.5	20.8
Rio de Janeiro	22.4	29.7	15.2	24.9	20	25.2	20
Nogales	16.5	34	-0.5	24.4	19.4	24.9	8.4
New Orleans	20.3	32.7	5.8	27.2	16.7	27.5	12.3
Miami	24.2	31.5	15.5	27	20	27.8	20

Temperature is estimated in $^{\circ}$C. Abbreviations: TempAnnual: mean annual temperature, maxTempWarmMonth: maximum temperature for the warmest month of the year, minTempColdMonth: minimum temperature for the coldest month of the year, meanTempWetQuar: mean temperature for the wettest quarter of the year, meanTempDryQuar: mean temperature for the driest quarter of the year, meanTempWarmQuar: mean temperature for the warmest quarter of the year, meanTempColdQuar: mean temperature for the coldest quarter of the year.

Table S4. Genetic diversity. Summary of the population genetic diversity statistics for the 56 populations of *Ae. aegypti* consisting the microsatellite dataset.

Population	Na	Ne	Ho	He	uHe	Fis	AR(30)
Luanda, Angola	6.17	3.40	0.62	0.67	0.68	0.07	5.36
Dakar, Senegal	4.92	2.62	0.61	0.57	0.57	-0.08	4.20
Bijagos, Gouinea-Bissau	6.42	3.59	0.60	0.65	0.66	0.09	5.70
Kichwamba, Uganda	6.08	3.77	0.62	0.69	0.70	0.10	5.58
N'goye, Senegal	6.50	3.84	0.64	0.69	0.70	0.07	5.53
Goudiry, Senegal	4.25	2.76	0.50	0.59	0.60	0.16	3.85
Sedhiou, Senegal	4.83	2.74	0.60	0.58	0.59	-0.03	4.34
Francesville, Gabon	7.17	4.02	0.62	0.70	0.71	0.10	6.15
Johannesburg, South Africa	5.92	3.33	0.62	0.63	0.64	<0.01	5.66
Maricopa, AZ, USA	4.33	2.52	0.54	0.56	0.57	0.05	3.95
Tijuana, Mexico	2.92	2.40	0.62	0.55	0.56	-0.13	2.91
Madera, CA, USA	3.92	2.62	0.53	0.54	0.55	0.02	3.60
Clovis, CA, USA	3.25	2.36	0.44	0.48	0.48	0.05	3.14
New Orleans, NO, USA	5.83	3.06	0.58	0.61	0.62	0.05	4.86
Cali, Colombia	4.17	2.25	0.46	0.52	0.53	0.15	3.56
Bolivar, Venezuela	4.25	2.61	0.55	0.56	0.57	<0.01	3.82
Cachoeiro, Brazil	4.00	2.24	0.46	0.49	0.50	0.09	3.50
Zulia, Venezuela	3.92	2.58	0.55	0.52	0.53	-0.09	3.65
Maraba, Brazil	4.08	2.47	0.53	0.56	0.57	0.04	3.64
Natal, Brazil	3.75	2.21	0.48	0.48	0.49	0.01	3.30
Miami, FLO, USA	5.42	3.45	0.65	0.63	0.63	-0.05	4.68
Las Palomas, Mexico	3.83	2.67	0.56	0.56	0.57	-0.02	3.57
Mazatan, Mexico	3.25	2.24	0.51	0.48	0.48	-0.05	3.14
Columbus, GA, USA	4.83	3.22	0.64	0.62	0.63	<0.01	4.32
Tapachula, Mexico	3.58	2.22	0.50	0.49	0.49	-0.02	3.27
Jacobina, Brazil	3.83	2.07	0.43	0.44	0.45	0.05	3.35
Nogales, Mexico	4.00	2.40	0.55	0.55	0.56	<0.01	3.70
Chetumal, Mexico	3.67	2.47	0.53	0.49	0.50	-0.06	3.48
Siquirres, Costa Rica	4.33	2.88	0.58	0.60	0.61	0.04	3.96
Rio de Janeiro, Brazil	4.42	2.15	0.42	0.48	0.49	0.11	3.89
Amacuzac, Mexico	4.00	2.87	0.55	0.55	0.56	<0.01	3.76
Cordoba, Argentina	4.08	2.56	0.56	0.56	0.58	0.03	3.75
Salta, Argentina	5.17	3.40	0.63	0.63	0.65	0.00	4.85
Key West, FLO, USA	4.92	3.18	0.62	0.62	0.63	0.01	4.37
Patillas, Puerto Rico	4.33	2.98	0.57	0.54	0.55	-0.07	4.03
Trinidad	4.17	2.66	0.47	0.53	0.53	0.15	3.87
Dominica	3.50	2.33	0.44	0.46	0.47	0.02	3.28
HCM, Vietnam	5.17	2.74	0.60	0.59	0.60	-0.02	4.46
Hanoi, Vietnam	3.42	2.09	0.46	0.47	0.48	0.09	3.20
Cebu City, Philippines	5.50	2.77	0.58	0.58	0.59	<0.01	4.66

Attock, Pakistan	4.75	2.94	0.52	0.59	0.59	0.12	4.31
Saudi Arabia	5.08	2.97	0.58	0.58	0.60	<0.01	4.53
Rayong, Thailand	4.33	2.48	0.56	0.55	0.56	<0.01	3.94
Prachuabkhirikan, Thailand	4.75	2.58	0.53	0.59	0.56	0.09	4.15
Townsville, Australia	3.83	2.63	0.51	0.55	0.56	0.07	3.63
Cairns, Australia	4.08	2.74	0.59	0.6	0.60	0.02	3.76
Bangkok, Thailand	4.67	2.84	0.59	0.59	0.60	-0.01	4.20
Madera, Portugal	3.58	2.43	0.48	0.54	0.55	0.12	3.30
Hawaii, USA	3.25	2.15	0.52	0.49	0.50	-0.07	3.12
Tahiti, French Polynesia	4.33	2.50	0.57	0.58	0.59	0.02	3.93
Rabai-out, Kenya	8.08	3.86	0.66	0.69	0.71	0.06	6.43
Nairobi, Kenya	7.75	3.61	0.60	0.68	0.70	0.11	6.20
Hopa, Turkey	3.17	2.32	0.50	0.52	0.54	0.03	3.10
Pazar, Turkey	3.08	2.00	0.53	0.47	0.48	-0.04	2.70
Coast, Georgia	3.00	2.16	0.50	0.51	0.52	<0.01	2.92
Inland, Georgia	2.67	2.00	0.42	0.45	0.46	0.05	2.67

Abbreviations: Na: total number of different alleles, Ho: observed Heterozygosity, He: expected Heterozygosity, uHe: Unbiased Expected Heterozygosity, Ne: Number of effective alleles, Fis: Fixation index, AR(30): allele richness estimated by rarefraction (N=30 genes).

Figure Legends

Figure S1. STRUCTURE bar plots based on the microsatellites dataset, including equal number of populations from each region. Population names are reported on their X axes. For each STRUCTURE run only the number of genetic clusters supported by the Evanno method is presented. For details see legend of Fig. 4.

Figure S2. STRUCTURE bar plots based on the microsatellites dataset, including equal number of *Ae. ae. aegypti* populations from each region. For each STRUCTURE run only the number of genetic clusters supported by the Evanno method is presented. For details see legend of Fig. 4.

Figure S3. Principal Components Analysis (PCA) on the global (a) and the *Ae. ae. aegypti* (b) microsatellite dataset as implemented and plotted using the ade4 package in R. Populations originated from different regions are presented with different colors. A barplot of the eigenvalues of the each PCA analysis is shown in the respective inset. *Abbreviations*: Aus, Australia; Pac, Pacific; Atl, Atlantic; BS, Black Sea.

Figure S4. Discriminant Analysis of Principal Components (DAPC) for the *Ae. ae. aegypti* populations based on the microsatellite dataset. The graph represents the individuals as dots and the groups as inertia ellipses. A barplot of eigenvalues for the discriminant analysis (DA eigenvalues) is displayed in the inset. The number of bars represent the number of discriminant functions that retained in the analysis and the eigenvalues correspond to the ratio of the variance between groups over the variance within groups for each discriminant function.

Figure S5. Discriminant Analysis of Principal Components (DAPC) for the *Ae. aegypti* populations collected from Turkey and Georgia based on the microsatellite dataset. For details see legend of Fig. S4.

Figure S6. Assignment test as implemented in Geneclass2 for the Black Sea populations and using the remaining worldwide populations as reference panel. The individual mosquitoes from Georgia and Turkey were assigned to the populations presented on the X axis of the barplots. The percentage of assignment for each one the Black Sea population to each one of the reference panel's population is presented on the Y axis.

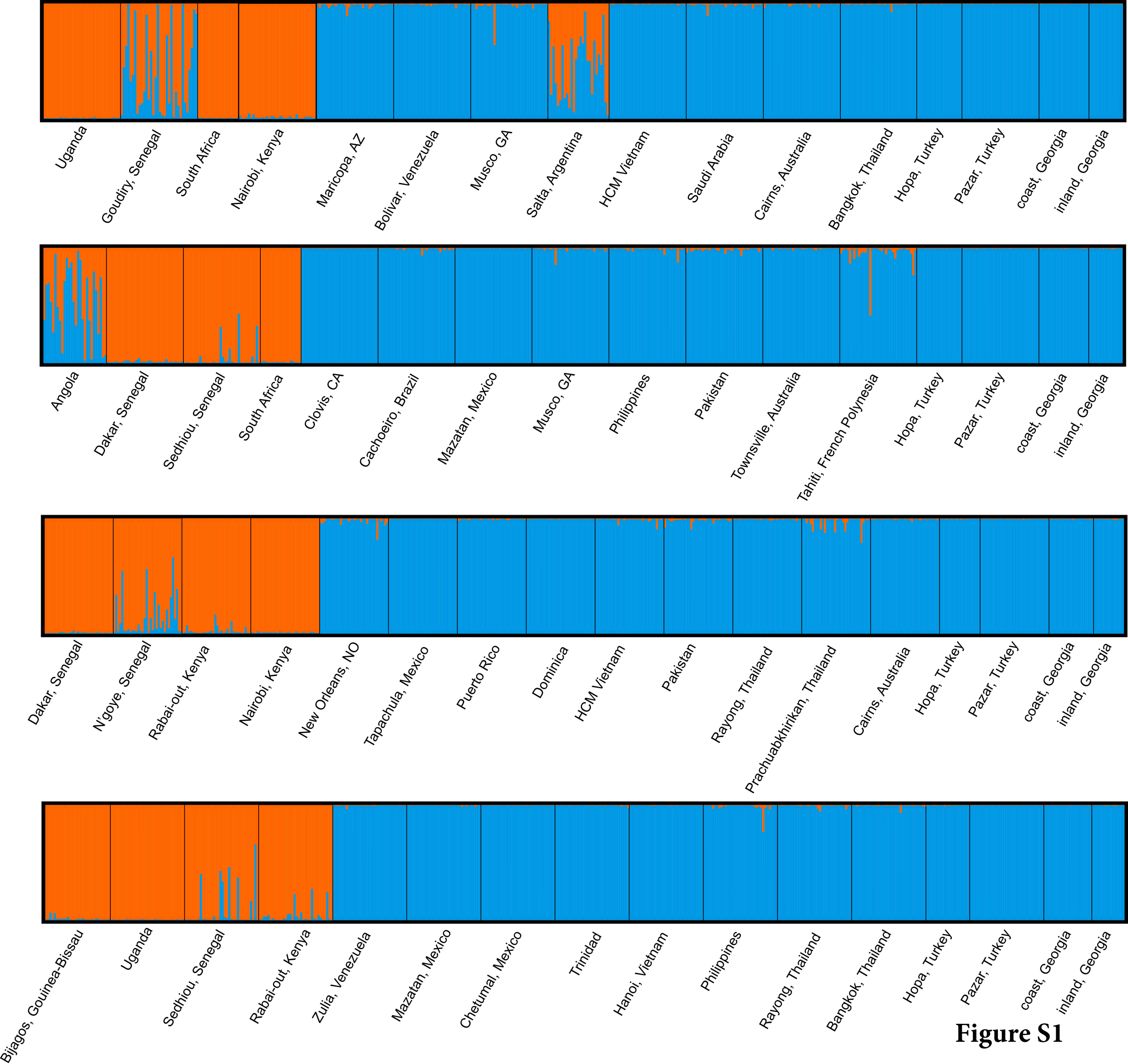

Uganda
Goudiry, Senegal
South Africa
Nairobi, Kenya
Maricopa, AZ
Bolivar, Venezuela
Musco, GA
Salta, Argentina
HCM Vietnam
Saudi Arabia
Cairns, Australia
Bangkok, Thailand
Hopa, Turkey
Pazar, Turkey
coast, Georgia
inland, Georgia
Angola
Dakar, Senegal
Sedhiou, Senegal
South Africa
Clovis, CA
Cachoeiro, Brazil
Mazatan, Mexico
Musco, GA
Philippines
Pakistan
Townsville, Australia
Tahiti, French Polynesia
Hopa, Turkey
Pazar, Turkey
coast, Georgia
inland, Georgia
Dakar, Senegal
N'goye, Senegal
Rabai-out, Kenya
Nairobi, Kenya
New Orleans, NO
Tapachula, Mexico
Puerto Rico
Dominica
HCM Vietnam
Pakistan
Rayong, Thailand
Prachuabkhirikan, Thailand
Cairns, Australia
Hopa, Turkey
Pazar, Turkey
coast, Georgia
inland, Georgia
Bijagos, Gouinea-Bissau
Uganda
Sedhiou, Senegal
Rabai-out, Kenya
Zulia, Venezuela
Mazatan, Mexico
Chetumal, Mexico
Trinidad
Hanoi, Vietnam
Philippines
Rayong, Thailand
Bangkok, Thailand
Hopa, Turkey
Pazar, Turkey
coast, Georgia
inland, Georgia

Figure S1

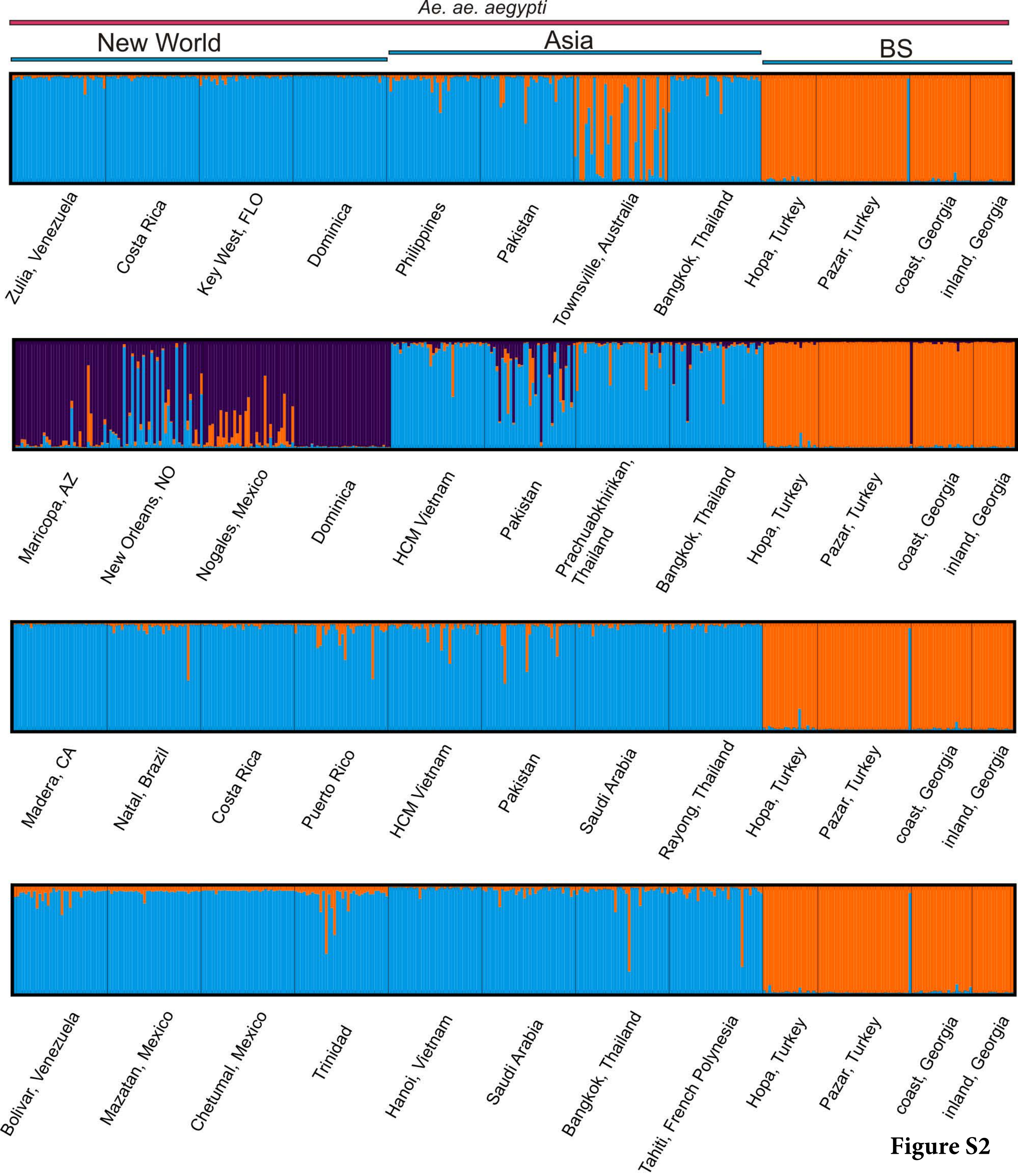
Ae. ae. aegypti
New World
Asia
BS
Zulia, Venezuela
Costa Rica
Key West, FLO
Dominica
Philippines
Pakistan
Townsville, Australia
Bangkok, Thailand
Hopa, Turkey
Pazar, Turkey
coast, Georgia
inland, Georgia
Maricopa, AZ
New Orleans, NO
Nogales, Mexico
Dominica
HCM Vietnam
Pakistan
Prachuabkhirikan, Thailand
Bangkok, Thailand
Hopa, Turkey
Pazar, Turkey
coast, Georgia
inland, Georgia
Madera, CA
Natal, Brazil
Costa Rica
Puerto Rico
HCM Vietnam
Pakistan
Saudi Arabia
Rayong, Thailand
Hopa, Turkey
Pazar, Turkey
coast, Georgia
inland, Georgia
Bolivar, Venezuela
Mazatan, Mexico
Chetumal, Mexico
Trinidad
Hanoi, Vietnam
Saudi Arabia
Bangkok, Thailand
Tahiti, French Polynesia
Hopa, Turkey
Pazar, Turkey
coast, Georgia
inland, Georgia

Figure S2

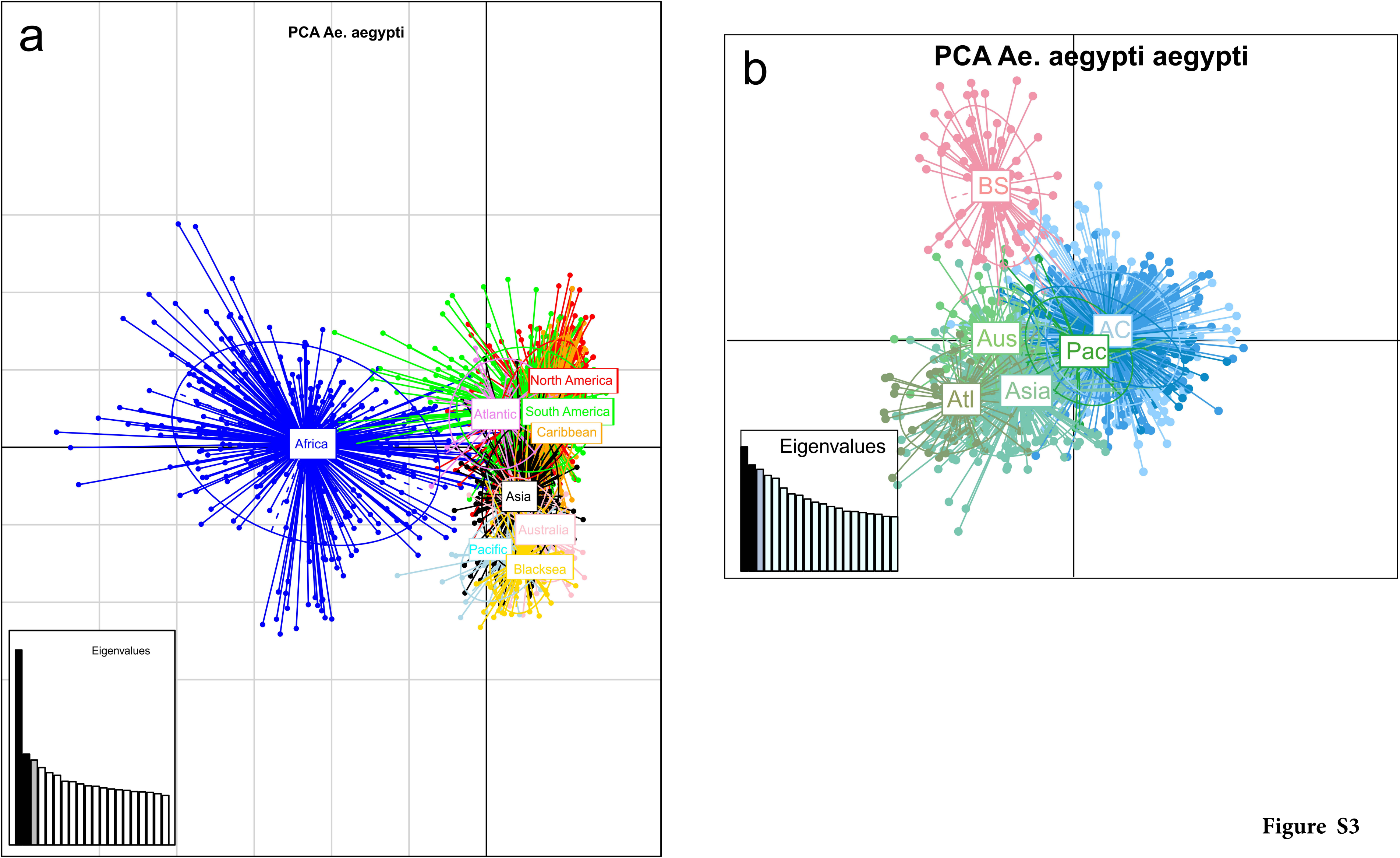

a
PCA Ae. aegypti
Africa
North America
Atlantic
South America
Caribbean
Asia
Australia
Pacific
Blacksea
Eigenvalues
b
PCA Ae. aegypti aegypti
BS
AC
Aus
Pac
Atl
Asia
Eigenvalues

Figure S3

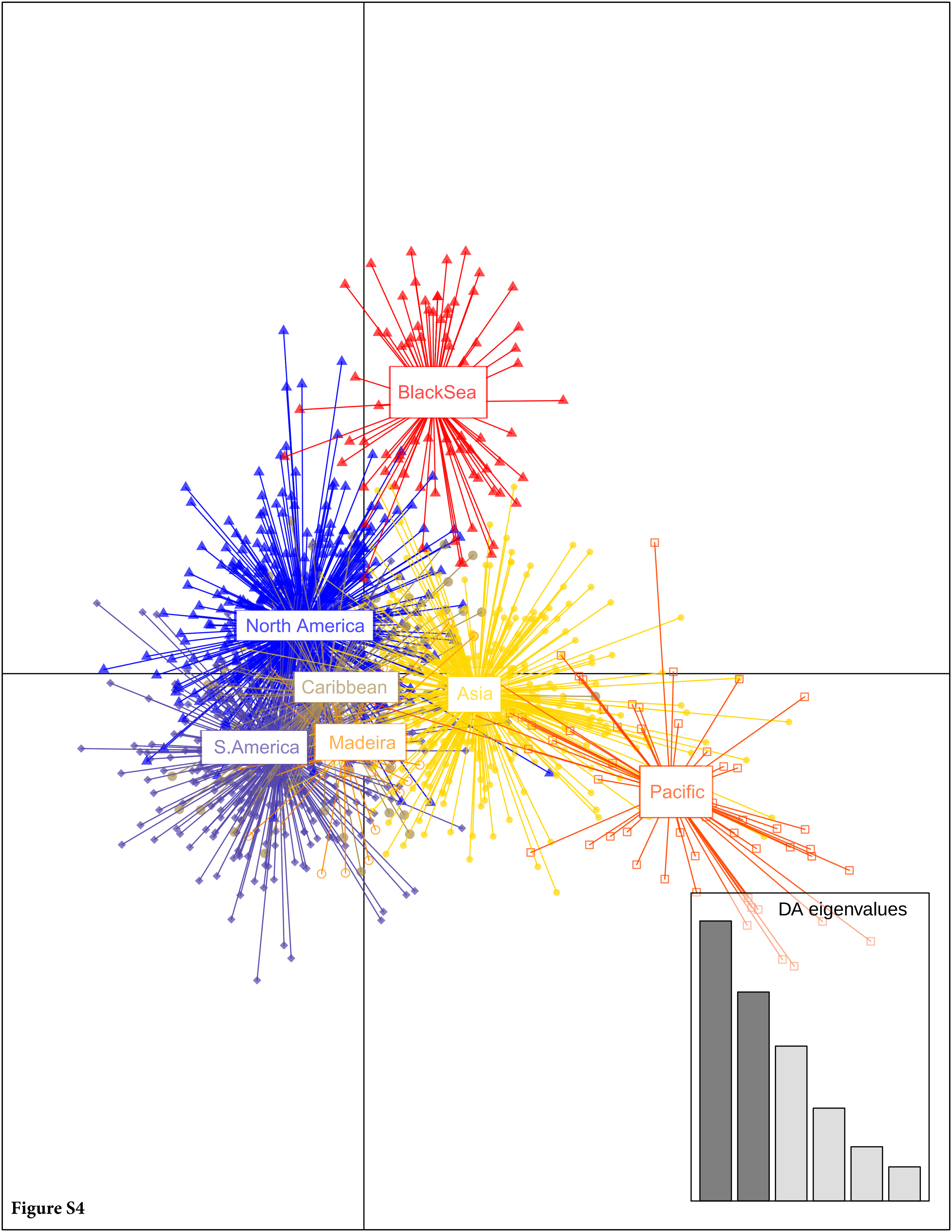

BlackSea
North America
Caribbean
Asia
S.America
Madeira
Pacific
DA eigenvalues

Figure S4

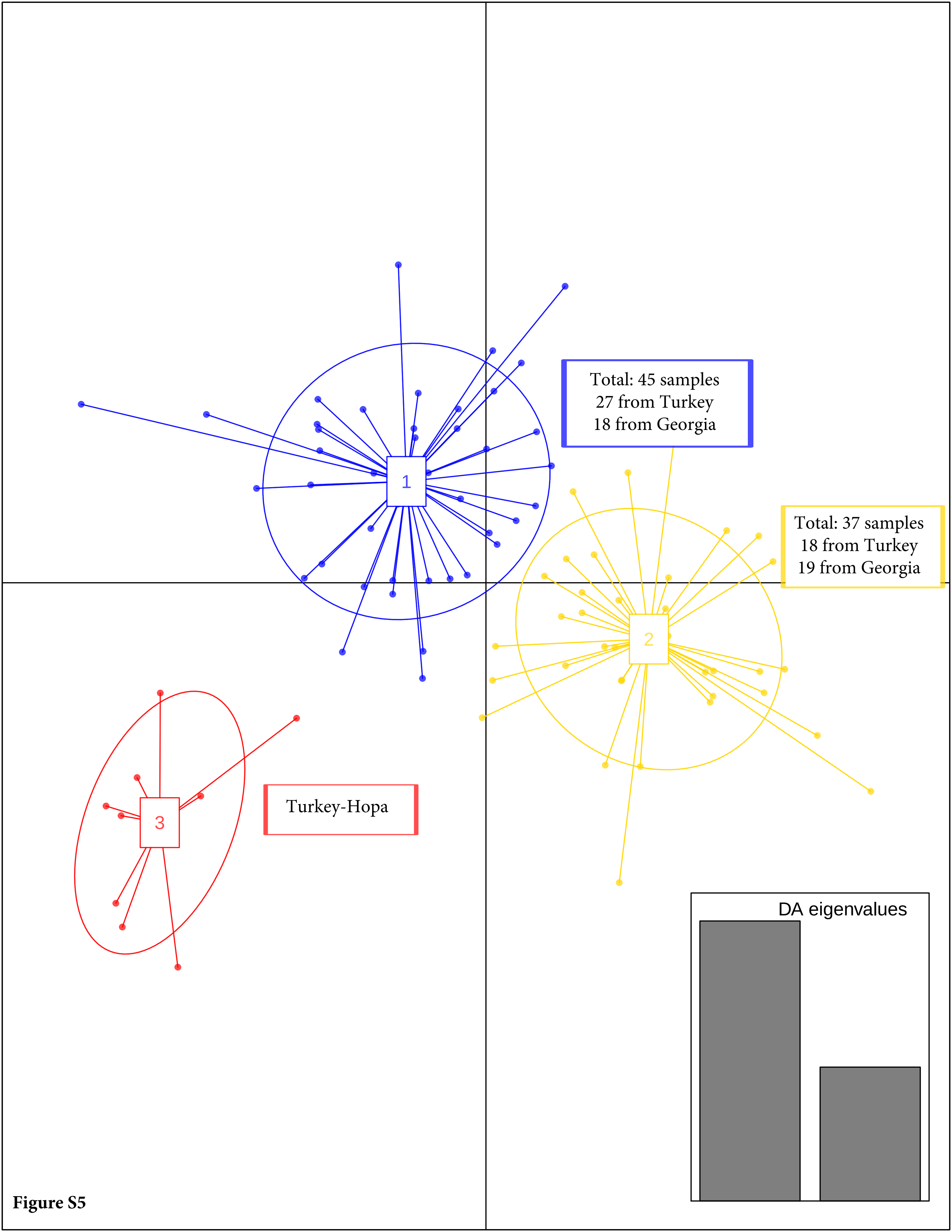
Total: 45 samples
27 from Turkey
18 from Georgia
Total: 37 samples
18 from Turkey
19 from Georgia
Turkey-Hopa
1
2
3
DA eigenvalues

Figure S5

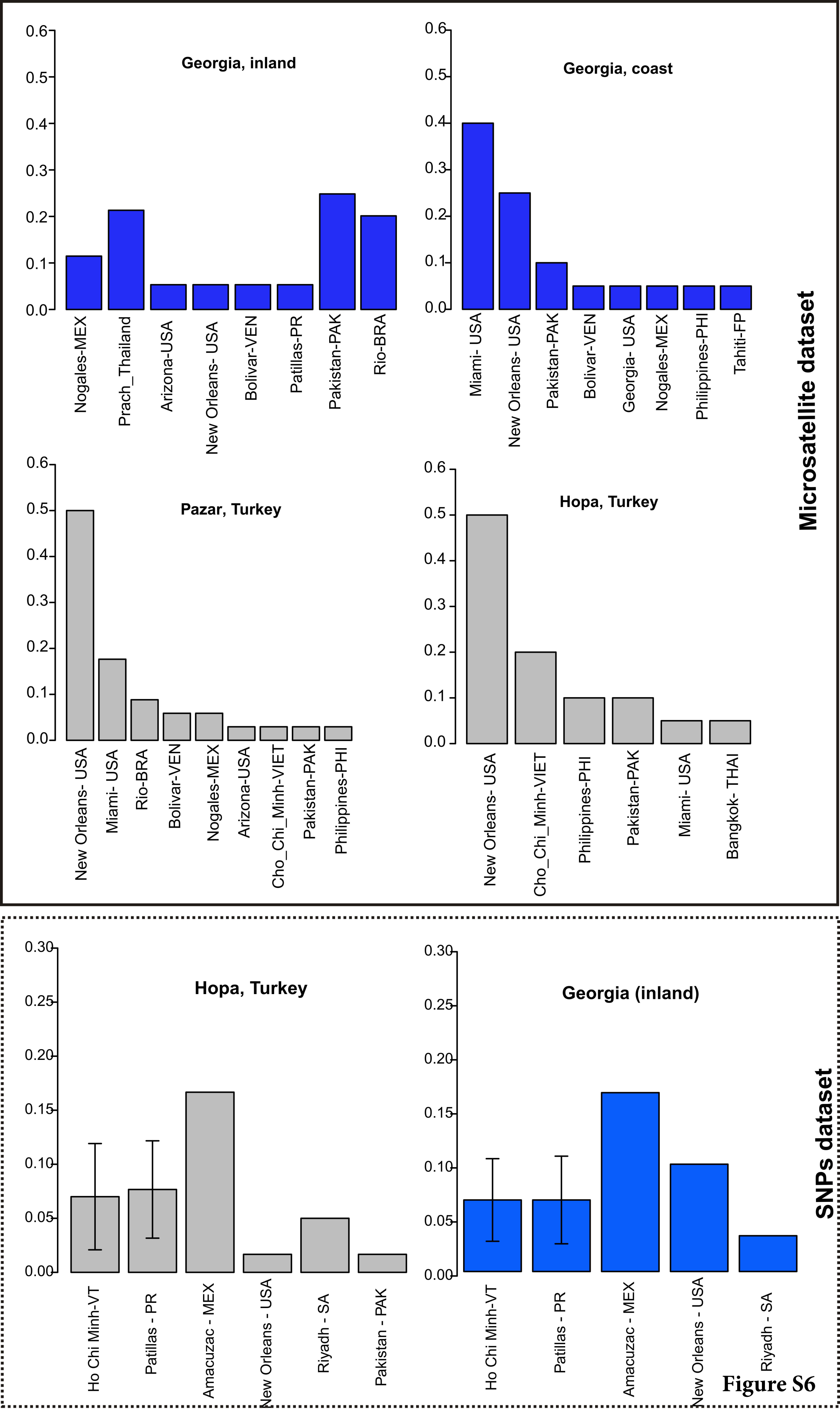
Microsatellite dataset
Georgia, inland
Nogales-MEX
Prach_Thailand
Arizona-USA
New Orleans- USA
Bolivar-VEN
Patillas-PR
Pakistan-PAK
Rio-BRA
Georgia, coast
Miami- USA
New Orleans- USA
Pakistan-PAK
Bolivar-VEN
Georgia- USA
Nogales-MEX
Philippines-PHI
Tahiti-FP
Pazar, Turkey
New Orleans- USA
Miami- USA
Rio-BRA
Bolivar-VEN
Nogales-MEX
Arizona-USA
Cho_Chi_Minh-VIET
Pakistan-PAK
Philippines-PHI
Hopa, Turkey
New Orleans- USA
Cho_Chi_Minh-VIET
Philippines-PHI
Pakistan-PAK
Miami- USA
Bangkok- THAI
SNPs dataset
Hopa, Turkey
Ho Chi Minh-VT
Patillas - PR
Amacuzac - MEX
New Orleans - USA
Riyadh - SA
Pakistan - PAK
Georgia (inland)
Ho Chi Minh-VT
Patillas - PR
Amacuzac - MEX
New Orleans - USA
Riyadh - SA

Figure S6
